# Organic Polyurethane Surface Structures to Manufacture Noneluting Antimicrobial Polyurethanes: A Systematic Review of Literature

**DOI:** 10.1155/bmri/6901867

**Published:** 2026-05-20

**Authors:** Bianca J. Hurck, Marian G. Vargas Guerrero, Candice de Boer, Aylvin A. Dias, Jacobus J. C. Arts

**Affiliations:** ^1^ Laboratory for Experimental Orthopedics, Department of Orthopaedic Surgery, Care and Public Health Research Institute (CAPHRI), Maastricht University Medical Centre, Maastricht, the Netherlands, mumc.nl; ^2^ DSM-Firmenich Biomedical, Geleen, the Netherlands; ^3^ Department Biomedical Engineering, Eindhoven University of Technology, Eindhoven, the Netherlands, tue.nl

**Keywords:** antiadhesion, bactericidal, bacteriostatic, chitosan, contact killing, guanidine, N-halamine, quaternary ammonium compounds, zwitterion

## Abstract

The dramatic increase in hospital‐acquired infections and healthcare costs associated with infection treatment and antimicrobial resistance in implant infections necessitates further research and development of antimicrobial implant material properties. Polyurethanes are key biomaterials used in medical implants and devices. A variety of compounds, both synthetic and natural, can be incorporated into polyurethanes to provide them with contact‐killing and/or bacteria‐repelling properties. This systematic literature review presents an up‐to‐date, comprehensive overview of organic, antimicrobial surface–active polyurethanes, the compounds used to impart them with antimicrobial properties, their effectiveness, and mode of action. Technologies preventing bacterial adherence, contact‐killing technologies, and environmental leaching are all analyzed. Highly effective compounds are reported but also compounds with low effectiveness. Overall, a genuine lack of standardization in methodological setup, outcomes, analysis, and reporting was discerned. Combined with ineffective technology, this hampers timely clinical implementation. Future research should focus on advancing the clinical efficacy while testing relevant models with high methodological quality, both in vitro and in vivo—bringing us one step closer to halting the rise of infection and antimicrobial resistance incidence by providing effective treatment technologies.

## 1. Introduction

Implants are integral to modern hospital care, and millions of patients are estimated to have received an implant worldwide, either temporary or permanent. Still, a significant portion of hospital‐acquired infections are related to these medical devices [1, 2]. An increasing number of implant infections are caused by bacteria resistance against antimicrobial compounds, which increases the danger of the infection at an alarming rate [3, 4]. A systematic analysis reported an estimated 4.95 million deaths associated with bacterial AMR in 2019 [5]. Furthermore, the rapid rise of antibiotic resistance means that 10 million people are projected to die annually from antimicrobial resistance in 2050, which might even be an underprediction [6, 7]. In comparison with other diseases, the predicted mortality rate is higher than cancer and diabetes combined, triggering the World Health Organization (WHO) to declare this the greatest threat to human and animal health [8].

Medical device–based infections are enabled by the formation of a bacterial biofilm, a common way bacteria shield themselves from antibiotics and the host′s immune reaction. These biofilms are notoriously difficult to clear from medical devices [4]. During biofilm formation, planktonic cells first attach to a surface [9, 10]. After adherence to the implant surface, bacteria build a protective extracellular matrix in the maturation stage composed of polysaccharides, proteins, nucleic acids (e‐DNA and e‐RNA), lipids, and other biomolecules [9–13]. Once the biofilm matures, cells start leaving it and form new biofilms in the dispersion stage [9, 10]. Biofilm can restrict the penetration of antibiotics when they bind to parts of the biofilm matrix or bacterial membranes or are inactivated by matrix enzymes [14]. It also hinders the immune system′s proper response. When biofilm forms on the implant surface within a couple of days, it is impossible to eradicate it in vivo. Therefore, technology development and treatment should be directed toward preventing bacterial adhesion on the implant surface, thereby impeding bacterial biofilm formation.

Common strategies for providing a material with antimicrobial properties are incorporating antibiotic agents into the material, coating the material with antibiotic agents that can elute, or modifying the surface to imbue a bacteriostatic or bactericidal effect [15]. In its strictest definition, “bactericidal” compounds kill bacteria and “bacteriostatic” compounds prevent their growth (the stationary phase of growth), but this applies only to laboratory environments [16]. According to the standard definition, antibiotics are classified as bactericidal if the ratio of the minimum bactericidal concentration (MBC) to the minimum inhibitory concentration (MIC) is ≤ 4, and as bacteriostatic if this ratio exceeds 4 [16]. However, these thresholds are somewhat arbitrary and do not consistently correlate with clinical outcomes in vivo [17]. Moreover, the distinction between bacteriostatic and bactericidal activity is not absolute; the same antimicrobial agent may exhibit bacteriostatic or bactericidal effects depending on the bacterial species tested and the concentration used [16]. In practice, bacteriostatic agents may still exert bactericidal effects under certain conditions, whereas bactericidal agents may not uniformly eliminate all bacterial populations [16].

Bacteriostatic and bactericidal surfaces can be divided into three groups: (1) surfaces that prevent bacteria from adhering to implant surfaces (antiadhesive), (2) surfaces that kill bacteria upon contact, and (3) surfaces containing leachable compounds that are toxic to bacteria [18]. Inorganic antibacterial compounds typically release ions, whereas organic compounds modify bacterial organelles and disrupt biochemical pathways. However, the mode of action varies depending on the specific antibacterial agent [19]. Polymers are the most employed material in medical device applications. It is estimated that 50% of all materials used in medical device manufacturing consist of plastic [20]. Various polymers are applicable to medical devices depending on their specific use, and polyurethane remains among the most popular and versatile synthetic biomedical polymers, in both composition and function [21, 22].

Polyurethanes are polymers formed by the reaction between diisocyanates and diols or polyols, which were first synthesized in 1937 [23, 24]. Since diols or polyols can be made with various chemical structures, their properties, such as mechanical strength, toughness, abrasion, chemical resistance, and degradation properties, are alterable. They can be used in various applications such as foams, coatings, binders, and adhesives [25, 26]. The polymer owes its popularity in medical devices to its tuneable chemical structures, excellent mechanical properties, predictable degradability, and biocompatibility [22, 27]. In recent years, scientific research has been focused on developing the ability to prevent microbial adhesion [15].

This systematic literature review is aimed at providing an overview and a critical appraisal of the antimicrobial properties of noneluting, organic polyurethanes exhibiting antimicrobial mechanisms. For this purpose, the review will elucidate what organic surface‐active agents and compounds are used with polyurethane technology and how successful they are in preventing bacterial adhesion and biofilm formation.

## 2. Methods

### 2.1. Protocol

This review is registered at Open Science Framework: https://osf.io/8tvaw/?view_only=31f70f7336204ea6952d710d2ee350b7.

**Figure 1:**
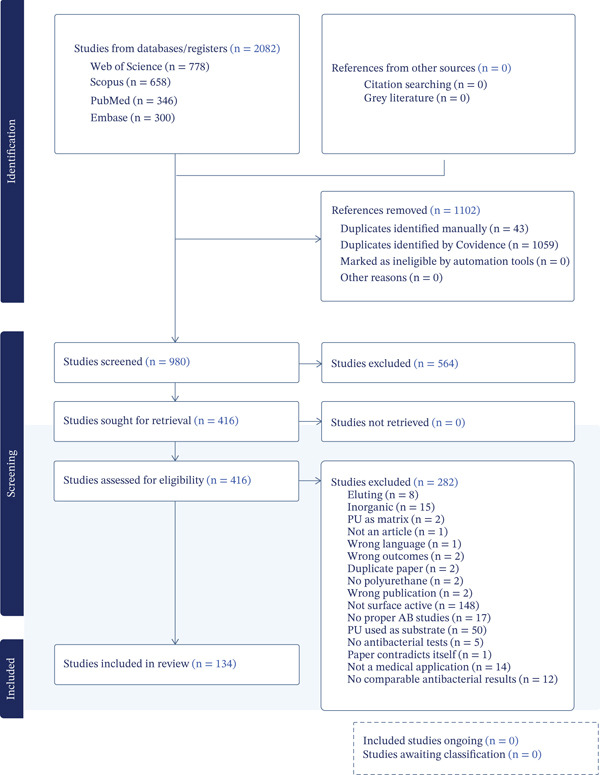
PRISMA flow chart.

The Preferred Reporting Items for Systematic Reviews and Meta‐Analyses (PRISMA) guidelines were used for this review [28]. As proposed in the guidelines, the PRISMA flowchart in Figure 1 summarizes the following steps, which were executed by two authors (B.J.H. and M.G.V.G.). Subsequently, any disagreements between the authors were solved by consensus with a third reviewer (C.A.). The search strategy is outlined in the Appendix: Supporting Information (Tables S1, S2, S3, S4, S5, S6, S7, S8, and S9).

### 2.2. Search Strategy

To identify the research articles that investigate antimicrobial polyurethanes, four different databases were searched: PubMed, Scopus, Web of Science, and Embase OVID. The results were limited to English scientific papers published between the years 2003 and February 2025. The following keyword combinations were used in the search: “Polyurethan∗” or “Polycarbamat∗” and “antibacterial” or “antimicrobial” and “Adhes∗” or “fouling” or “Contact‐killing” or “Contact” or “Drug‐Eluting” or “Elut∗.” To concentrate on the medical applications of antimicrobial polyurethanes, the keywords (“marine” or “packaging” or “waste water”) were excluded.

### 2.3. Study Selection

First, the studies were imported for screening and, after excluding all duplicates, the rest of the studies were further screened. Data excluded were as follows:

1. all studies that were explicitly about nonmedical applications,
2. all articles about materials that were not based on polyurethanes,
3. all articles concentrating on antifungal or antiviral mechanisms, and
4. all articles without in vitro or in vivo antibacterial experiments. After the first screening process, it was decided that
5. to further narrow the scope of this review, solely eluting systems are excluded for the following reasons: (1) There is a wide variety of antimicrobial compounds and ions that can be formulated into polyurethanes. (2) A contact killing approach is believed to be complementary to the widely practiced use of antibiotics for prophylaxis. (3) Eluting systems are anticipated to further enable antimicrobial resistance due to their persistence in the environment [18, 29]. Furthermore, this mechanism is also known to have a negative influence on the environment [18]. Considering the rising antibiotic resistance in the population, avoiding polyurethanes that elute antibiotics could also be advantageous. Compounds reported to exhibit both surface activity and elution will be included in the analysis. Finally, after writing the first draft, it was decided that (6) all studies involving polyurethanes containing inorganic compounds will be removed from this review, including all polyurethanes containing metals, graphene, inorganic silicates, and silicone. Antimicrobial inorganic compounds are likely to have a different mode of action than organic compounds, and focusing on one type of compound further narrows the scope of the review. The hereby excluded studies can be investigated further in a subsequent review.

The studies were selected in two steps: First, only the title and abstract of all studies were screened according to the above‐explained criteria (by B.J.H. and M.G.V.G.). Secondly, the full text of all papers was assessed for eligibility (Figure 1).

### 2.4. Data Extraction and Analysis

Finally, to obtain information about the characteristics important to this study, one reviewer (B.J.H.) extracted the data, and a second reviewer (M.G.V.G.) verified 15% of the studies. In case of disagreement, a third reviewer (C.d.B.) was consulted. The extracted data includes matrix, material, modification, mode of action, microorganism, inoculum size, broth, testing method, inhibition remark, and information on cytotoxicity. The extracted data is summarized in Tables S1, S2, S3, S4, S5, S6, S7, S8, and S9 (Appendix: Supporting Information) as well as in Tables 1, 2, 3, 4, 5, 6, 7, 8, 9, 10, 11, 12, 13, 14, 15, 16, and 17.

**Table 1:** Extracted information—Miscellaneous polyurethanes. Abbreviations used: N A = not assessed; T = tested;  ^“^/^”^ = information not provided.

Author	Year	Material	Modification	AM category	Microorganism	Testing method/broth	Inhibition remark/inoculum	Cytotoxicity	In vivo
Uscategui et al. [30]	2016	Polyurethanes synthesized with castor oil	Polycaprolactone (backbone)	Contact killing (bacteriocidal)	*E. coli* (ATCC 2469) and *P. aeruginosa* (ATCC 27853) (4.2 *x* 10^6^ and 4.8 *x* 10^6^ *C* *F* *U*/*m* *L*, respectively)	Colony counting (Trypticase soy broth)	77% (log 0.63) reduction for *E. coli* and 56% (log 0.35) reduction for *P. aeruginosa* after 24 h	No	N A
Uscategui et al. [31]	2019	Polyurethanes synthesized with castor oil polyols	Long aliphatic side segments (pendant)	Contact killing (bacteriocidal)	*E. coli* and *P. aeruginosa* (/)	Turbidity measurements (Trypticase soy broth)	Bacterial inhibition: 7.5% *E. coli* and 6.9% *P. aeruginosa* (< 1 log)	No	T (wound healing)
Kultys and Puszka [32]	2014	Poly(thiourethane‐urethane)	One‐step melt polymerization from poly(oxytetramethylene) diol or poly(hexamethylene carbonate) diol of as soft segments (no specific AM groups)	/	*S. epidermidis* (ATCC 12228) or *E. coli* (ATCC 25922) (150 *x* 10^6^ *C* *F* *U*/*m* *L*)	Turbidity measurements (turbidity nutrient broth)	*S. epidermis* control: bacterial reduction 64.4% *E. coli* bacterial reduction 6.7% (< 1 log)	N A	N A

**Table 2:** Extracted information—Halogen‐containing compounds. Abbreviations used: N A = not assessed; T = tested;  ^“^/ ^”^ = information not provided.

Author	Year	Material	Modification	AC category	Microorganism	Testing method/broth	Inhibition remark/inoculum	Cytotoxicity	In vivo
No ZOI									
Sun et al. [33]	2009	Polyurethane	N‐chlorinated alkoxy‐s‐triazines (blend)	Contact killing (bacteriocidal)	*S. aureus* (ATCC 6538) and *E. coli* (ATCC 15597), (antimicrobial activities: 10 *μ*L of 10^8^–10^9^ CFU/mL, biofilm activities: 5 mL of 10^7^–10^8^ CFU/mL)	Colony counting, Kirby–Bauer test, SEM (tryptic soy broth) for *S. aureus*, and (Luria–Bertani) broth for *E. coli*	6% active chlorine content: All the samples tested provided a total kill of *S. aureus* and *E. coli* within 5 min (6‐ to 7‐log reduction), no zone of inhibition.	N A	N A
Luo et al. [34]	2006	Polyurethane	N‐halamine‐based polymeric additive, poly[(6‐morpholino‐s‐triazine‐2,4‐diyl)‐N‐chloro‐[2,2,6,6‐tet‐ramethyl‐4‐piperidyl)imino]‐hexamethylene[(2,2,6,6–4‐piperidyl) imino)] (blend)	Contact killing (bacteriocidal)	*S. epidermidis* RP62A (ATCC 35984) (10 *μ*L of 10^6^–10^7^ CFU/mL)	Colony counting and Kirby–Bauer test (trypticase soy broth)	Total kill of the bacteria after 120 min (4‐ to 5‐log reduction), no zone of inhibition.	N A	N A
ZOI									
Xiu et al. [35]	2017	Polyurethane/N‐halamine semi‐interpenetrating polymer network	N‐halamine, N‐chloro‐2, 2, 6, 6‐tetramethyl‐4‐piperidyl methacrylate (blend)	Contact killing and elution (bacteriocidal)	Antimicrobial activity: *S. epidermidis* (ATCC 35984) and *A. calcoaceticus* (ATCC 31926) (2 *μ*L of 10^8^–10^9^ CFU/mL) Kirby–Bauer test: *A. calcoaceticus* (ATCC 31926) (10^8^–10^9^ CFU/mL), antifouling activity of the N‐halamine semi‐IPN: *S. epidermidis* and *A. calcoaceticus* (10^5^–10^6^ CFU/mL)	Colony counting, Kirby–Bauer test, and SEM (/)	After 4 h of contact, all the semi‐IPNs provided a total kill of *A. calcoaceticus* and *S. epidermidis* (5‐ to 6‐log reduction), a small, yet clear zone against the bacteria.	N A	N A
ZOI not tested									
Lin et al. [36]	2015	Polyurethane	Fluorinated N‐halamine, 1‐chloro‐3‐1H,1H,2H,2H‐perflurooctyl‐5,5‐dimetylhydantoin (Cl‐FODMH), and its unfluorinated counterpart, 1‐chloro‐3‐octyl‐5,5‐dimethylhydantoin (Cl‐ODMH) (blend)	Contact killing and antiadhesion (bacteriocidal)	*S. epidermidis* (ATCC 35984) and *A. calcoaceticus* (ATCC 31926) (2 mL of 10^8^–10^9^ CFU/mL)	Colony counting and SEM (Nutrient broth solution)	At higher than 3% Cl‐ODMH content, no recoverable bacteria (8‐ to 9‐log reduction) could be isolated; but even with 5% of Cl‐FODMH, (2.58 ± 1.14) *x* 10^2^ *C* *F* *U*/*c* *m* ^2^ of *A. calcoaceticus* were recovered from the film (5 log for Cl‐ODMH and 3 log for Cl‐FODMH after 15 min).	N A	N A
Tan and Obendorf [37]	2007	Polyurethane	N‐halamine (made from 2,2,5,5‐tetramethyl‐imidozalidin‐4‐one hydantoin) (pendant)	Contact killing (bacteriocidal)	*E. coli* (ATCC 13706) and *S. aureus* (ATCC 6538) (25 *μ*L of 2–4 × 10^8^ *C* *F* *U*/*m* *L*)	(AATCC) test method 100–2004: colony counting (/)	Total kill after 2 h contact (6 log).	N A	N A
Bisquera and Sumera [38]	2011	Polyurethane	N‐halamine derivative: N‐Hydroxymethylated Hydantoin (terminal)	Contact killing (bacteriocidal)	*S. aureus* and *E. coli* (7.5 *x* 10^9^ *C* *F* *U*/*m* *L*) and *C. albicans*	AATCC‐100‐1999: colony counting (/)	A complete kill after 2‐h challenge time with *E. coli* (4‐log reduction) and a log reduction of 3.30 and 3.70 for *S. aureus* and *E. coli*, respectively, was observed with a 0.626 log reduction for *C. albicans* (yeast).	N A	N A
Xiu et al. [39]	2017	Polyurethane semi‐interpenetrating polymer networks	N‐halamine precursor, 3‐(4 ^′^‐vinylbenzyl)‐5,5‐dimethylhydantoin (blend)	Contact killing (bacteriocidal)	*S. epidermidis* and *A. calcoaceticus* (2.5 *μ*L of 10^8^–10^9^ CFU/mL for colony counting and 10^5^−106 CFU/mL for SEM)	Colony counting and SEM (nutrient broth)	Total kill of the testing bacteria after 30 min (6‐ to 8‐log).	N A	N A
Barnes et al. [40]	2007	Polyurethane	Amine monomer 4‐[3‐triethoxysilylpropoxyl]‐2,2,6,6‐tetramethylpiperidine (pendant)	Contact killing and elution (bacteriocidal)	*S. aureus* (ATCC 6538) (4.67 *x* 10^4^ *C* *F* *U*), *E. coli* O157: H7 (ATCC 43895) (1.87 *x* 10^3^ *C* *F* *U*)	Colony counting (/)	After 3 h: 4.67‐log reduction for *S. aureus* and 3.27‐log reduction for *E. coli* O157: H7.	N A	N A
Liang et al. [41]	2007	Polyurethane	3‐(3‐triethoxysilylpropyl)‐7,7,9,9‐tetramethyl‐1,3,8‐triaza spiro‐[4.5]decane‐2,4‐dione treated with hypochlorite to make N‐halamines (pendant)	Contact killing (bacteriocidal)	*S. aureus* (ATCC 6538) and *E. coli* (O157:H7 ATCC 43895) (10^3^–10^5^ CFU)	Colony counting (/)	TS‐Cl formulation 9.61 × 10^16^ *a* *t* *o* *m* *s*/*c* *m* ^2^ Cl^+^ with 2‐h contact and 5.88 log red for *S. aureus* and 3.38 reduction for *E. coli* O157:H7.	N A	N A
Grunzinger et al. [42]	2007	Polyurethane polymer surface modifiers with hydantoin‐containing soft blocks	Hydantoin‐containing polymer surface modifiers (blend) react with hypochlorite to generate the biocidal chloramide moiety	Contact killing (bacteriocidal)	*P. aeruginosa* (PAO1) and *S. aureus* (ATCC‐25904) (10^7^–10^8^ CFU/mL)	AATCC‐100: colony counting and aerosol spray test (Luria broth)	4.38‐log reduction after 30 min.	N A	N A
Yin et al. [43]	2021	Thermoplastic polyurethane and hydrophilic modified polyacrylic acid blend	Blend of 2,2,6,6‐tetramethyl‐4‐piperidinol grafted PAA (gPAA) with TPU	Contact killing and elution (bacteriocidal)	*S. aureus* and *E. coli* (10^6^ CFU/mL)	Colony counting, turbidity assay, SEM, dehydrogenase activity assay (/)	> 95% reduction after 5 min (> 1.3 log).	N A	N A
Raut et al. [44]	2018	Polyurethane/polyvinylpyrrolidone iodine (PU/PVPI) blends	Polyvinylpyrrolidone iodine (blend)	Contact killing and antiadhesion (bacteriocidal)	*S. aureus* (MCC 2408), *S. epidermidis* (NCIM 2493), and *P. aeruginosa* (NCIM 5029) (1 *x* 10^6^ cell suspension)	Colony counting, SEM, and live/dead staining (tryptone soy broth)	After 24 h of incubation, bacterial adhesion on PU/PVPI‐1.5% showed around 99% (2 log) reduction for *S. aureus* and *S. epidermidis*, whereas PU/PVPI‐1.5% showed 90% (1 log) reduction for *P. aeruginosa*.	N A	N A

**Table 3:** Summary of log‐reduction values reported in studies of polyurethane materials functionalized exclusively with quaternary ammonium compounds (QACs) categorized according to the location of the QACs within the polymer architecture (pendant, backbone, terminal, or multiple positions).

	Pendant	Backbone	Terminal	Backbone+terminal	Backbone+pendant
< 1 log	One study	Two studies	/	One study	/
1 log	Three studies	/	/	/	/
2 log	One study	One study	/	/	/
3 log	Five studies	Three studies	/	/	/
4 log	One study	/	One study	/	/
5 log	Five studies	/	/	/	/
6 log	Two studies	/	/	/	/
7 log	One study	/	/	/	One study

Abbreviation: /, no applicable studies found.

**Table 4:** Extracted information—Quaternary compounds. Abbreviations used: N A = not assessed; T = tested;  ^“^/ = information not provided.

Author	Year	Material	Modification	AM category	Microorganism	Testing method/broth	Inhibition remark/inoculum	Cytotoxicity	In vivo
No ZOI									
Wanget al. [45]	2020	Waterborne polyurethane.	Quaternary ammonium salt (pendant).	Contact killing (bacteriocidal)	*S. epidermidis* (ATCC 12228) and *Escherichia coli* (ATCC 25922) (10^5^ cells/mL)	Colony counting, Kirby–Bauer test (Luria–Bertani broth)	5‐log reduction (total kill) against both bacteria, no zone of inhibition.	N A	N A
Yagci et al. [46]	2011	Polyurethane.	Quaternary ammonium compounds (pendant).	Contact killing (bacteriocidal)	*Staphylococcus aureus* and *E. coli* (10^5^ bacteria/mL)	ISO 22196: Colony counting, Kirby–Bauer test (Luria–Bertani broth)	5‐log reduction of both bacterial species observed at all concentrations applied, no zone of inhibition.	N A	N A
Chen et al. [47]	2017	Polyurethane acrylate.	Quaternary ammonium methacrylate compounds bearing hydrophobic tails with different alkyl chain lengths (QACs as pendant).	Contact killing (bacteriocidal)	*S. epidermidis* (ATCC 25922) and *E. coli* (ATCC 12228 [10^5^ CFU/mL])	Colony counting, Kirby–Bauer test, sterile (Luria–Bertani broth)	5‐log reduction (total kill), no zone of inhibition.	N A	N A
Bakhshi et al. [48]	2012	Polyurethane coatings from functional soybean oil with a polyol with pendant dimethylphenylammonium iodide groups modification.	Quaternary ammonium functions (QACs as pendant).	Contact killing (bacteriocidal)	Kirby–Bauer: *E. coli* (1 *x* 10^4^ *C* *F* *U*), turbidity measurements: *E. coli* or *S. aureus* (1.5 *x* 10^4^ CFU)	Kirby–Bauer test and turbidity measurements (Luria–Bertani broth)	Bacterial reduction: *E. coli*: 99.4% (2.2 log), *S. aureus*: 100% (4 log); no zone of inhibition was observed for *E. coli*, whereas a considerable zone of inhibition was detected for *S. aureus* (up to 12.3 mm).	No	N A
Yari et al. [49]	2012	Polyurethane.	Poxy‐functional quaternary ammonium compound: glycidyltriehtylammonium chloride (terminal).	Contact killing (bacteriocidal)	Kirby–Bauer: *E. coli* (1.0*x*10^4^ *C* *F* *U*), turbidity measurements: *S. aureus* and *E. coli* ( 1.5*x*10^4^ *C* *F* *U* CFU)	Kirby–Bauer test and turbidity measurements (Luria–Bertani broth)	Bacterial reduction: 100% for *E. coli* and *S. aureus* (4 log), no zone of inhibition.	No	N A
Hu et al. [50]	2019	Polyurethane.	Side‐chain quaternized (pendant).	Contact killing (bacteriocidal)	*E. coli* (DMS 1077) (1 × 10^6^ *C* *F* *U*/*m* *L*)	Turbidity measurements, Kirby–Bauer test methods using DMS 1077 (nutrient‐broth solution)	Prevented the formation of at least 99.9% (3 log) of the daughter cells for longer than 48 h, no zone of inhibition.	N A	N A
Lee et al. [51]	2020	Thermoplastic polyurethane	Nanoclay with quaternary ammonium salt groups (blend).	Contact killing (bacteriocidal)	*E. coli* J53 (KACC 16628) and *S. aureus* subsp. *aureus* (KACC 10768) (colony counting: 10^7^ CFU/mL; Kirby−Bauer: 10^4^ CFU/mL)	Colony counting, Kirby−Bauer test (Mueller−Hinton broth)	98.5% (1.8 log) killing efficiency against Gram‐negative *E. coli* and 99.9% (3‐log) against Gram‐positive *S. aureus*, no zone of inhibition.	N A	N A
Singh et al. [52]	2024	Tetracopolymer thermoplastic polyurethane	Quaternary ammonium compounds (pendant).	Contact killing (bacteriocidal)	Visually analyzed *E. coli* (1000 CFU/mL); Kirby−Bauer *E. coli*, *S. aureus*, and *C. albicans* (100 mL of 1000 CFU), prolonged antimicrobial activity study *E. coli*, *S. aureus*, and *C. albicans* (1000 CFUs); endurance of antimicrobial activity *E. coli* (1000 CFU/mL); biofilm formation assay coculture of *E. coli*, *S. aureus*, and *C. albicans* (10^5^ CFU/mL)	Antimicrobial potential study: visually analyzedKirby−Bauer test, colony counting, FESEM (field emission scanning electron microscope) model JSM‐IT700HR, JOEL (Japan) (Luria–Bertani broth)	Contact killing for 180 days with > 99% (> 2 log) efficacy, the coating retained antimicrobial properties after 75 wash cycles with 50% ethanol, no zone of inhibition.	No	N A
Bakhshi et al. [53]	2013	Polyurethane.	Quaternary ammonium compounds (pendant).	Contact killing (bacteriocidal)	Kirby–Bauer: *E. coli* (1 × 10^4^ *C* *F* *U*), turbidity measurement: *E. coli* or *S. aureus* (1.5 × 10^4^ *C* *F* *U*)	Kirby–Bauer test, turbidity measurement (Luria–Bertani broth)	Bacteria reduction: 88.7% (< 1 log) *E. coli* and 95.4% (1.3 log) *S. aureus*, no zone of inhibition.	No	N A
Liu et al. [54]	2015	Polyurethane.	Carbamate group‐containing quaternary ammonium salts (pendant).	Contact killing (bacteriocidal)	*S. aureus* and *E. coli* (minimum inhibitory concentration test: 10^6^ CFU/mL colony counting: 5 × 10^5^–1 × 10^6^ *C* *F* *U*/*m* *L*, Kirby–Bauer: 1 × 10^4^ CFU [only *E. coli*])	Minimum inhibitory concentration test, colony counting, Kirby–Bauer test (Luria–Bertani media)	*E. coli*: 55.0% and *S. aureus*: 45.2% bacteria reduction (1 log), no zone of inhibition.	N A	N A
Borahet al. [55]	2022	Thermoplastic polyurethane cationomers.	Quaternary ammonium groups (backbone).	Contact killing (bacteriocidal)	*S. aureus* (MTCC‐96), *E. coli* (MTCC‐443), and *Pseudomonas aeruginosa* (MTCC‐74) (10^6^ CFU/mL)	Kirby–Bauer test, turbidity test (nutrient broth)	Growth inhibition: 29% *E. coli* and 28% *S. aureus* (< 1 log); no clear zone of inhibition.	N A	N A
Wang R. et al. [56]	2016	Polyurethane.	Quaternary ammonium salts (backbone).	Contact killing and antiadhesion (bacteriocidal)	*E. coli* or *S. aureus* (Kirby–Bauer: 10^7^ CFU/mL, turbidity measurement: 10^6^ CFU/mL, live/dead staining: 10^6^ CFU/mL)	Kirby–Bauer test, turbidity measurement, live/dead staining (/)	*E. coli* after 6 h: 6.7% bacterial reduction and *S. aureus* after 8 h: 25% bacterial reduction (< 1 log), no zone of inhibition.	N A	N A
ZOI									
Hua and Odelius [57]	2017	Polyhydroxyurethane.	Quaternary ammonium compounds and sulfonium compounds with iodine counter ions (backbone).	Contact killing and Elution (bacteriocidal)	*E. coli* and *S. aureus* (≈3 × 10^6^ *C* *F* *U*)	Kirby–Bauer test and turbidity measurements (Luria–Bertani media)	The QAC‐loaded films exhibit outstanding bactericide properties (> 99.9%, > 3 log), and the antibacterial mechanism is demonstrated to be a dual killing mechanism, with zones of inhibition if not conditioned in LB media (swollen sample).	N A	N A
Wang et al. [58]	2016	Polyurethane.	Quaternary ammonium salt N‐methyl‐N‐dodecyl‐N, N‐bis(2‐hydroxyethyl) ammonium bromide (backbone and terminal).	Contact killing and elution (bacteriocidal)	*E. coli*, *S. aureus*, and *B. subtilis* (/)	Kirby–Bauer test and colony counting (/)	The antibacterial ratios of TAPU against *E. coli*, *S. aureus*, and *B. subtilis* were 89.7%, 81.2% and 72.1%, respectively, (< 1 log), zone of inhibition with a diameter up to 26.3 mm for *E. coli.*	N A	N A
Rabiee et al. [59]	2024	Polyurethane prepolymer.	A hydroxyurethane functionalized with methacrylate and quaternary ammonium groups (pendant).	Contact killing and elution depending on concentration (bacteriocidal)	*S. aureus*, *E. coli* (/)	Shaking flask method under dynamic contact condition, Kirby–Bauer test (/)	LMTSO40: Bacterial reduction of 36% and 27% for *S. aureus* and *E. coli*, respectively, (< 1 log), and no zone of inhibition, LMTSO60: Bacterial reduction of 66% and 47% for *S. aureus* and *E. coli* (< 1 log), respectively, and a visual zone of inhibition.	No	N A
									
Tran et al. [60]	2015	Polyurethane.	Poly diallyl‐dimethylammonium chloride bonded to PU dressing by “dehydration bonding” (pendant).	Contact killing (bacteriocidal)	*S. aureus*, *Pseudomonas aeruginosa*, and *A. baumannii* (colony counting: 10^3^ CFU/mL, SEM: 10^8^ CFU/mL)	Colony counting, confocal laser scanning microscopy and SEM (Luria–Bertani broth or Mueller–Hinton broth)	Complete killing (3 log) was observed in less than 5 min for *S. aureus*; however, complete killing took a longer time for *P. aeruginosa* (< 2 h) and *A. baumannii* (< 1 h); no biofilm was recovered.	N A	N A
Zhan et al. [61]	2017	Waterborne biodegradable polyurethanes.	Lysine‐derivative gemini quaternary ammonium salt chain extenders with different hydrophobic alkyl chain lengths (pendant).	Contact killing and antiadhesion (bacteriocidal)	*E. coli* (ATCC 25922), *S. aureus* (ATCC 6538) (10^6^–10^7^ CFU/mL)	Broth microdilution method, colony counting, TTC staining (Mueller–Hinton broth)	No bacterial colonies were observed on their surface after 6 h (6‐log reduction).	No	N A
Gharibi et al. [62]	2019	Polyurethane.	Quaternary ammonium salt containing fatty amide molecules with reactive siloxane moieties (pendant).	Contact killing (bacteriocidal)	Methicillin‐resistant *S. aureus* (ATCC 33593), *P. aeruginosa* (ATCC 9027), and *C. albicans* (ATCC 10231) (1 mL of 2 × 10^8^ *C* *F* *U*/*m* *L*)	Colony counting (tryptic soy broth)	100% killing activity (8 log) against all the studied strains after 24 h.	No	N A
Gharibi et al. [63]	2022	Polyurethane–siloxane.	Quaternary ammonium moieties (pendant).	Contact killing (bacteriocidal)	*E. coli* and *B. subtilis* (2 × 10^8^ *C* *F* *U*/*m* *L* ‐ > diluted 1:100 and 100 *μ*L used)	ASTM E 2180‐07/colony counting (tryptic soy broth)	100% reduction (5 log) after 24 h.	No	N A
Wu and Hsu [64]	2016	Waterborne biodegradable cationic polyurethane.	Quaternary ammonium, tertiary ammonium groups in the backbone and side chain.	Contact killing (bacteriocidal)	*E. coli* and *S. aureus* (1 mL of 1 × 10^7^ *C* *F* *U*/*m* *L*)	ASTM E2315‐03: Colony counting (nutrient broth)	Nanoparticles: 100% inhibition (7 log) with a contact time of 3 h against both bacteria. WCPU films: Antibacterial rate of *E. coli* and *S. aureus* reached 100% after 24 h of contact.	No	N A
Gao et al. [65]	2023	A monomer containing a temperature‐sensitive N‐isopropyl amide derivative and pH‐sensitive tertiary amine groups are copolymerized with a polyurethane chain, and partial tertiary amine groups are quaternized.	Quaternized amine groups (pendant).	Contact killing and antiadhesion (bacteriocidal)	*S. aureus* (CICC 10 384) (10^6^ CFU/mL)	Fluorescence inverted microscope, TESCAN MIRA3 field emission scanning electron microscope (tryptic soy broth)	Up to 7‐log reduction.	No	N A
Zander et al. [66]	2018	Thermoplastic polyurethane containing an allyl ether side‐chain functionality.	A series of quaternary ammonium thiol compounds possessing various hydrocarbon tail lengths were attached to the surface using thiolene “click” chemistry (pendant).	Contact killing (bacteriocidal)	*S. epidermidis* (ATCC 12228), *S. aureus* (25923), *E. coli* (ATCC 25922), *P. aeruginosa* (ATCC 27853), *E. faecalis* (ATCC 29212), and methicillin‐resistant *S. aureus* (ATCC BAA‐41) (8.5 *x* 10^5^ *C* *F* *U*/*m* *L*)	ISO 22196: Colony counting, live/dead staining, and SEM (tryptic soy broth)	A 6‐log reduction (99.9999%) in *E. coli* and complete reductions (5 log) in *S. epidermidis* were observed for all Qx‐SH compositions.	No	N A
Lin et al. [67]	2023	A biodegradable bilayer polyurethane fibrous membrane with two sides with Janus properties.	Bioactive molecule dopamine and antimicrobial gemini quaternary ammonium salt (pendant).	Contact killing (bacteriocidal)	*S. aureus* (ATCC 25923) and *S. mutans* (ATCC 35668) (10^6^ CFU/mL)	Colony counting(brain heart–leachate medium)	Antibacterial efficiency of up to 3 log.	No	N A
Lv et al. [68]	2024	Thermoplastic polyurethanes.	Two types of quaternary ammonium salts (QAS)–containing chain: (1) N‐methyl‐N‐alkyl‐N,N‐bis(2‐hydroxyethyl) ammonium bromide (backbone) and (2) N,N‐dimethyl‐N‐alkyl‐N‐2,3‐propylene glycol (pendant).	Contact killing (bacteriocidal)	*S. aureus* (minimum inhibitory concentration assay: 10^6^ CFU/mL and field emission scanning electron microscopy: 50 *μ*L of 5 × 10^5^ *C* *F* *U*/*m* *L*)	Minimum inhibitory concentration assay and Field emission scanning electron microscopy (Mueller–Hinton medium)	Remarkably, almost 100% (3 log) of *S. aureus* could be killed.	No	N A
Yao et al. [69]	2008	Electrospun polyurethane.	Quaternized poly(4‐vinyl‐N‐hexylpyridinium bromide (pendant).	Contact killing (bacteriocidal)	*S. aureus* and *E. coli* (bacterial adhesion: 10^7^ cells/mL, colony counting: 10^6^ cells/mL)	SEM and colony counting (3.1% yeast–dextrose broth)	High antibacterial efficacy for *S. aureus* reaching 99.9% (3 log) and 99.999% (5 log) after 1‐and 4‐h contact, respectively. In comparison, the antibacterial efficacy for *E. coli* was 99.9% (3 log) after 4 h of contact.	N A	N A
Peng et al. [70]	2023	Zwitterionic polyurethane polymers were dipcoated onto a thermoplastic polyurethane substrate.	Quaternary amine groups (backbone).	Contact killing (bacteriocidal)	*E. coli* and *S. aureus* (/)	Live/dead staining and colony counting (/)	Bactericidal ratios of the coatings against *E. coli* and *S. aureus* reached 99.9% (3 log).	No	Reoperative infection model in the subcutaneous tissue of SD rats
Pan et al. [71]	2025	Polyurethane.	Different contents of quaternary ammonium salt groups in the PU main chain and two vinyl end groups.	Contact killing (bacteriocidal)	*E. coli*, *P. aeruginosa*, and *S. aureus* (10^6^ CFU/mL)	ISO 22196 standard/colony counting and SEM (/)	Rapidly inactivating 72.8% (> 1 log), 99.9%, (3 log), and 98.9% (2.0 log) of *E. coli*, *S. aureus*, and *P. aeruginosa* within 30 min.	N A	N A
Sun et al. [72]	2024	Imidazolium‐based ionic thermoplastic polyurethane elastomers.	Imidazole salt diols with different alkyl chain lengths at the N‐position of imidazolium cation (QAC in pendant).	Contact killing (bacteriocidal)	*S. aureus* and *E. coli* (/)	Minimum inhibitory concentration measurement, colony counting (/)	An antibacterial rate of more than 99.9% (3 log) against both *S. aureus* and *E. coli*, the antibacterial activity could be restored by washing dead bacteria off.	N A	N A
Tapia et al. [73]	2024	Nonisocyanate polyurethanes (linear and crosslinked).	Quaternary ammonium containing cyclocarbonate oligomer (backbone).	Contact killing (bacteriocidal)	*S. aureus* (ATCC 29213), *S. epidermidis* (ATCC 35984), and *P. aeruginosa* (PA14) (10^5^ and 10^6^ CFU/mL)	Microdilution method, colony counting (“brain heart infusion” nutritive medium)	Inhibition of bacterial adhesion of 99.9% (3 log) for all bacteria, no growth was observed on any of the plates after incubation; after 24 h of incubation, no living bacteria were observed in either the film or the solution.	No	N A
Li, et al. [74]	2022	Cationic waterborne polyurethane.	Quaternary ammonium salt (pendant).	Contact killing (bacteriocidal)	*E. coli* (ATCC 25922) and *S. aureus* bacteria (ATCC 29213) (/)	Colony counting (/)	85.03% (< 1 log) and 99.46% (2.3 log) bacterial reduction, respectively.	N A	N A
Zhang et al. [75]	2023	Nonionic plant oil–based nonisocyanate waterborne poly(hydroxyl urethane).	Carbonated linseed oil (CLSO) and quaternary ammonium salt (backbone).	Contact killing (bacteriocidal)	*S. aureus* (ATCC 29213) and *E. coli* (ATCC 25922) (10^7^ CFU/mL)	Colony counting (/)	Antibacterial efficiency toward *S. aureus* and *E. coli* above 98% (1.7‐log) for all films.	N A	N A
He et al. [76]	2022	Polyurethane.	Integrating bioactive dopamine and an antibacterial gemini quaternary ammonium salt (pendant).	Contact killing (bacteriocidal)	*S. aureus* (ATCC 25923) and *S. mutans* (ATCC 35668), (10^6^ CFU/mL)	Colony counting (brain heart infusion medium)	Excellent antibacterial efficiency above 90% (1 log) on both *S. aureus* and *S. mutans*.	N A	N A

**Table 5:** Extracted information—Guanidine. Abbreviations used: N A = not assessed; T = tested;  ^“^/^”^ = information not provided.

Author	Year	Material	Modification	AM category	Microorganism	Testing method/broth	Inhibition remark/inoculum	Cytotoxicity	In vivo
Peng et al. [77]	2017	Polyurethane	Click‐suitable pentasubstituted guanidine (2‐propinyl‐1,1,3,3‐tetramethylguanidine) (pendant)	Contact killing (Bacteriocidal)	*E. coli* (ATCC 25922) and *S. aureus* (ATCC 29213) (10^5^ CFU/mL)	Colony counting and Kirby–Bauer test (/)	Around 99.9% (3 log) killing of *S. aureus* and 98.0% (1.7‐log) killing of *E. coli*; no zone of inhibition.	N A	N A
Zhanget al. [78]	2021	Anionic and cationic waterborne polyurethane	Type B Poly(hexamethylene guanidine hydrochloride) (blend)	Contact killing (bacteriocidal)	*S. aureus* and *E. coli* (1–10 × 10^5^ *C* *F* *U*/*m* *L*)	ISO 22196: colony counting, Kirby–Bauer test (tryptic soy broth)	The antibacterial efficiency can reach more than 99% (2 log); no zone of inhibition.	N A	N A
Richards et al. [79]	2014	Certofix (hydrophilic polymer, blended with polyurethane, and a polyhexanide functionalized with a methacrylate group)	Polyhexanide functionalized with a methacrylate group (blend)	Antiadhesion (bacteriostatic)	*S. aureus*, *S. epidermidis*, *P. aeruginosa*, *A. baumannii*, *K. pneumoniae*, and *C. albicans* (2 × 10^6^ *C* *F* *U*/*m* *L*)	Turbidity measurements, colony counting, crystal violet staining (tryptic soy broth)	*S. aureus*: 1.4‐log reduction, 96% after 60 min; *S. epidermidis*: similar to *S. aureus*; *P. aeruginosa*: 0.5‐log reduction, 67% after 60 min; *A. baumannii*: total kill 6‐log reduction after 60 min; *K. pneumoniae*: 1.2‐log reduction, 94% after 60 min.	N A	N A
Gharibi and Agarwal [80]	2021	Uretdione containing thermoplastic polyurethane	Polyguanidine bactericidal agent (pendant)	Contact killing and antiadhesion (bacteriocidal)	*E. coli* and *B. subtilis* (/)	Colony counting and SEM (/)	Preventing the formation of at least 99.9% (3 log) of the daughter cells.	No	N A

**Table 6:** Extracted information—Other cationic compounds. Abbreviations used: N A = not assessed; T = tested;  ^“^/^”^ = information not provided.

Author	Year	Material	Modification	AM category	Microorganism	Testing method/broth	Inhibition remark/inoculum	Cytotoxicity	In vivo
PEI									
Gultekinoglu et al. [81]	2015	Polyurethane	Brush‐like polyethyleneimine and alkylated polyethyleneimine (pendant)	Contact killing and antiadhesion (bacteriocidal)	*K. pneumoniae* (ATCC 21523), *E. coli* (ATCC25922) and *P. mirabilis* (1 *x* 10^7^ *C* *F* *U*/*m* *L*)	Colony counting (/)	Resistance to adhesion against Gram‐negative bacterial species up to two orders of magnitude.	No	N A
Gultekinoglu et al. [82]	2017	Polyurethane	Cationic polyethyleneimine brushes grafted on PU (pendant)	Contact killing and antiadhesion (bacteriocidal)	*P. mirabilis* (ATCC 29906) (1 × 10^8^ *C* *F* *U*/*m* *L*)	Dynamic biofilm reactor system (tryptic soy broth)	Decreased the biofilm formation up to two orders of magnitude after 24 h.	N A	T (wound healing)
Gultekinoglu et al. [83]	2016	Polyurethane	Grafted cationic polyethyleneimine brushes (pendant)	Contact killing and anti‐adhesion (bacteriocidal)	*E. coli* (K‐12) (/)	Single‐cell force spectroscopy (SCFS) (Luria–Bertani medium)	Alkylated high molecular weight PEI brushes show the lowest rupture force (56.1 pN) with the lowest binding percentage (5%) against single bacterial cells.	N A	N A
**Boron**									
Sürdem, et al. [84]	2022	Polyurethane	Boric acid (backbone)	Contact killing and antiadhesion (bacteriocidal)	*E. hirae* (ATCC 10571), *S. aureus* (ATCC 25923), *P. aeruginosa* (ATCC 27853) and *E. coli* (ATCC 25922) (10^6^ CFU/mL)	AATCC 100‐2004: colony counting (Mueller–Hinton broth)	*E. hirae*: 4.6 log, 99.998% reduction; *S. aureus*: 4.8 log, 99.998% reduction; *P. aeruginosa*: 3.9 log, 99.988% reduction; *E.coli*: 3.5 log, 99.968% reduction.	N A	N A
**Nonionic**									
Sehmi et al. [85]	2016	Polyurethane.	Glutaraldehydeimpregnated (blend).	Contact killing (bacteriocidal)	*S. aureus* (8325‐4) and *E. coli* (ATCC 25922) (25 *μ*L of ~10^6^ CFU/mL)	Colony counting (brain heart infusion broth)	The number of *S. aureus* was reduced to below the detection limit of 100 CFU/mL (≥ 4 log; *p* < 0.001).	N A	N A
Wang et al. [86]	2012	Polyurethane.	Three types of surfactants (cationic, nonionic, and anionic), surfactant‐modified random nanosilicate platelets (blend).	Contact killing and elution (bacteriostatic)	*S. aureus* and *E. coli* (0.4 mL of 1 × 10^6^ *C* *F* *U*/*m* *L*)	Colony counting (nutrient broth)	100% (5 log) bacterial reduction for all materials.	No	T (wound healing)
Peng et al. [87]	2018	Polyurethane	Pendant benzisothiazolinone (PU‐BIT) connected using click chemistry	Contact killing (bacteriocidal)	*E. coli* (ATCC 25922) and *S. aureus* (ATCC 29213) (10^5^ CFU/mL)	Colony counting and Kirby–Bauer test (Mueller–Hinton broth medium)	A bactericidal efficacy of 91.6% (1.1 log) and 30% (< 1 log) against *S. aureus* and *E. coli*, respectively,no zone of inhibition.	N A	N A

**Table 7:** Extracted information—Chitosan. Abbreviations used: N A = not assessed; T = tested;  ^“^/^”^ = information not provided.

Author	Year	Material	Modification	AM category	Microorganism	Testing method/broth	Inhibition remark/inoculum	Cytotoxicity	In vivo
Liu, et al. [88]	2016	Polyurethane.	Citric acid and chitosan were covalently immobilized on polyurethane (pendant).	Contact killing (bacteriocidal)	*P. aeruginosa* (ATCC 10145) (1.5 × 10^5^ *c* *e* *l* *l* *s*/*m* *L*)	Colony counting (nutrient broth)	Complete kill after 18 h (over 5‐log reduction).	No	N A
Yang et al. [89]	2008	Hydroxyl‐terminated polybutadiene–based polyurethane modified with N‐isopropyl acrylamide.	Chitosan (blend).	Contact killing (bacteriocidal)	*P. aeruginosa* and *S. aureus* (10^9^ CFU/mL)	Colony counting (/)	99.99997% (6.5‐log) *S. aureus* reduction and 99.99998% (6.7‐log) *P. aeruginosa* reduction.	No	N A
Uscátegui, et al. [90]	2017	Polyurethane‐based bioadhesive synthesized from polyols derived from castor oil.	Chitosan (blend).	Contact killing (bacteriocidal)	*E. coli* (ATCC 2469) (1.17 *x* 10^7^ *C* *F* *U*/*m* *L*)	Colony counting (tryptic soy broth)	*E. coli* decreased 60%–90% (0.4‐1 log) after 24 h.	No	N A
Xiong et al. [91]	2024	PLA‐COL@PU‐COL@PLA‐PU‐COL, polylactic acid (PLA), polyurethane (PU), collagen (COL).	Heparin (not used for AM testing) or chitosan (pendant).	Contact killing (bacteriocidal)	*S. aureus* and *E. coli* (10^6^ CFU/mL)	Colony counting method (LB nutrient agar, broth medium)	The antibacterial rate against *E. coli* and *S. aureus* was 60.38*%* ± 3.17*%*(<1 log) and 82.14*%* ± 3.85*%*(<1 log), respectively.	No	N A
Bahrami et al. [92]	2019	Polyurethane was synthesized using castor oil and a trade‐grade of hexamethylene diisocyanate.	Chitosan or collagen (pendant).	Contact killing (bacteriocidal)	*S. aureus* (ATCC 6538, PTCC 1112) and *E. coli* (ATCC 25922, PTCC 1399) (1.5 × 10^8^)	Colony counting (/)	Collagen‐modified: *E. coli* 44% (0.25 log reduction), *S. aureus* 74% (0.41‐log reduction); chitosan‐modified: *E. coli* 60.4% (0.59‐log reduction), *S. aureus* 68% (0.49‐log reduction).	No	N A
Ren et al. [93]	2023	Photocurable polyurethane.	Catechol groups modified oligochitosan (blend).	Contact killing (bacteriocidal)	*E. coli* (ATCC 8739) and *S. aureus* (ATCC 6538) (1 *x* 10^5^ *C* *F* *U*/*m* *L*)	Colony counting (Luria–Bertani)	Highest antibacterial rate against *E. coli*, at 15.79%, for *S. aureus*, best antibacterial rate was at 36.36% and 54.91% depending on the composition (< 1 log).	N A	N A
Yu et al. [94]	2006	Waterborne polyurethanes (with ester or ether soft segments).	6‐O‐carboxymethylchitosan semi‐interpenetrating polymer network (blend).	Contact killing (bacteriocidal)	*E. coli* (10^6^ CFU/mL)	Colony counting (nutrient broth)	0.21‐log reduction, 37.6% reduction.	N A	N A
Kara et al. [95]	2014	Polyurethane films were synthesized from toluene diisocyanate and polypropylene ethylene glycol.	The film surfaces were modified by covalent immobilization of chitosan (pendant).	Contact killing and antiadhesion (bacteriocidal)	*S. aureus* and *P. aeruginosa* (10^7^–10^8^ CFU/mL)	Colony counting and SEM (nutrient broth)	*P. aeruginosa*: 4.7‐log reduction, *S. aureus*: 5‐log reduction	N A	N A
Arevalo et al. [96]	2016	Polyurethanes with 5% *w/w* of polycaprolactone.	Chitosan as an additive in different concentrations (0.5, 1.0, and 2.0% *w*/*w*) (blend).	Contact killing and antiadhesion (bacteriocidal)	*E. coli* (ATCC 2469) and *S. aureus* (ATCC 6538) (4.2 *x* 10^6^ CFU/mL)	Colony counting (tryptic soy broth)	*S. aureus*: 54% bacteria reduction (0.3‐log reduction), *E. coli*: 36% bacteria reduction (0.3‐log reduction).	No	N A

**Table 8:** Extracted information—Other natural compounds. Abbreviations used: N A = not assessed; T = tested;  ^“^/^”^ = information not provided.

Author	Year	Material	Modification	AM category	Microorganism	Testing method/broth	Inhibition remark/inoculum	Cytotoxicity	In vivo	
Chitin										
Zia et al. [97]	2013	Hydroxy‐terminated polybutadiene–chitin–based polyurethanes.	Chitin (terminal).	Contact killing, antiadhesion and elution (bacteriocidal and bacteriostatic)	*E. coli* (/)	Kirby–Bauer test (/)	Diameter without zone: 22 mm and diameter with zone: 30 mm	N A	N A	
Peptides										
Lu et al. [98]	2021	Thermoplastic polyurethane.	Thermoplastic polyurethane surface modification of peptide polymer using plasma surface activation and substitution reaction between thiol and bromide groups (pendant).	Contact killing (bacteriocidal)	Methicillin‐resistant *S. aureus* (USA 300), *S. haemolyticus* (R01), *E. coli* (JM109), and *Pseudomonas aeruginosa* (9) (5 × 10^5^ *C* *F* *U*/*m* *L*)	Colony counting, leaching assay (fluorescence), SEM (/)	Showing killing efficacy of 99.9% (3 log) against *E. coli*, 97.5% (1.6 log) against MRSA, 99.9% (3 log) against *S. haemolyticus*, and 91.2% (1.1 log) against *P. aeruginosa*, respectively, no leaching	No	Subcutaneous implantation infectious model with (SD) rats	Methicillin‐resistant *Staphylococcus aureus* (5 × 10^5^ *C* *F* *U*/*m* *L*), colony counting with a 1.27‐log reduction
Peng et al. [99]	2018	Tecoflex, a commercial thermoplastic polyurethane.	Peptide‐like cationic pendant functional groups.	Contact killing (bacteriocidal)	*E. coli* (25 922) (/)	Live–dead staining, colony counting, Kirby–Bauer test, and SEM (M9 minimal medium)	Excellent antibiofilm properties against *E. coli* even after 5 days.—no zone of inhibition —no effect on planktonic bacteria.	No	N A	
Curcumin										
Abdelbasset et al. [100]	2023	Polyurethane.	Curcumin (blend).	Contact killing (bacteriocidal)	*S. aureus*, and *E. coli* (/)	Turbidity measurement (Mueller–Hinton medium)	Antibacterial efficiency: *E. coli*: 95.2% (1.3 log); *S. aureus*: 93.7% (1.2 log).	No	N A	
Other compounds										
Dhyani et al. [101]	2024	Polyurethane.	Two different EO components: alpha‐terpineol and cinnamaldehyde (terminal).	Contact killing and elution (bacteriocidal)	*E. coli*, methicillin‐resistant *S. aureus*, and *P. aeruginosa* (~10^6^ CFU/mL)	Colony counting (tryptic soy broth)	Up to 6‐log bacterial reduction	N A.	In vivo full‐thickness porcine burn model	*S. aureus* 10^7^ CFU/mL with > 5‐log reduction on Days 2 and 3
Taş et al. [102]	2022	Hybrid waterborne polyurethane/polydopamine matrix.	Covalently immobilized lysostaphin (pendant).	Contact killing (bacteriocidal)	*S. aureus* (ATCC 29213) (antibacterial properties: 10^5^ CFU/mL, antibiofilm properties: 10^8^ CFU/mL)	ISO 22196: Colony counting, SEM, and LSCM (tryptic soy broth)	Strong anti‐*S. aureus* activity with a 4‐log reduction	N A	N A	
Yang et al. [103]	2024	Poly(oxime‐urethane) synthesized with isophorone diisocyanate and poly(tetramethylene glycol).	Biobased chain extender, 2,5‐diformylfuran dioxime (pendant).	Contact killing (bacteriocidal)	*E. coli* (CICC 10389) and *S. aureus* (CICC 10384) (600 *μ*L of 10^5^ CFU)	Colony counting (soya casein digest lecithin polysorbate broth)	100% (4 log) reduction of *E. coli* and *S. aureus*	N A	N A	
Sen et al. [104]	2021	Polyurethane.	*Antharaea mylitta* silk–fibroin (blend).	Contact killing (bacteriocidal)	*E. coli* (ATCC 25922), *S. aureus* (ATCC 6538), *P. aeruginosa* (ATCC 15442), *K. pneumoniae* (ATCC 6538) (1 *x* 10^8^ *C* *F* *U*/*m* *L*)	Turbidity measurement, lactate dehydrogenase activity assay and Kirby–Bauer test (nutrient broth)	*E. coli*: 31% reduction *S. aureus*: 53% reduction*P. aeruginosa*: 70% reduction *K. pneumoniae*: 58% reduction (> 1 log); biggest ZOI: 1.85 cm	No	N A	
Grover et al. [105]	2016	Polyurethane.	Immobilization of acylase from *Aspergillus melleus* (blend).	Contact killing (bacteriocidal)	*P. aeruginosa* ATCC 10145 and PAO1 (10^4^ CFU/mL)	Colony counting (Luria–Bertani broth)	60% and 58% (> 1 log) reduction in biofilm formation by *P. aeruginosa* strains ATCC 10145 and PAO1, respectively	N A	N A	
Tomaselli et al. [106]	2023	Water‐blown polyurethane was prepared by biopolyols from epoxidized linseed oils and caprylic acid in combination with toluene diisocyanate.	A series of terpenes (menthol, geraniol, terpineol, and borneol) was included in the starting formulations (blend).	Contact killing (bacteriocidal)	*E. coli* (ATCC 11229) and *S. aureus* (ATCC 6538) (1.5 × 10^5^–3.0 × 10^5^ *C* *F* *U*/*m* *L*)	Colony counting (peptone for *E. coli* and brain heart infusion for *S. aureus*)	Reducing Gram+ and Gram− viability by more than 60% (> 1 log)	N A	N A	

**Table 9:** Extracted information—Surface modifications. Abbreviations used: N A = not assessed; T = tested;  ^“^/^”^ = information not provided.

Author	Year	Material	Modification	AM category	Microorganism	Testing method/broth	Inhibition remark/inoculum	Cytotoxicity	In vivo
Siddiquie et al. [107]	2020	Polyurethane elastomer	Surface micro‐/nanotexturing	Antiadhesion (bacteriocidal)	*E. coli* (BL21 [DE3])(1 × 10^8^ *C* *F* *U*/*m* *L*)	Spinning disk confocal microscope (Luria–Bertani broth)	Reduction in bacterial adhesion: 96.1% (1.4 log), respectively.	N A	N A
Ganbaatar et al. [108]	2025	Polyurethane acrylate	Nanoline array with a width and height of 0.5 *μ*m, and spacing of 0.3, 0.5, 1, 2, and 4 *μ*m	Contact killing and antiadhesion (bactericidal or bacteriostatic depending on the spacing)	*E. coli* (ATCC 25404) and *S. aureus* (ATCC 25923) (/)	Colony counting, SEM, and confocal microscopy analyses (Luria–Bertani broth)	Up to 79% bacterial reduction (< 1 log).	N A	N A
May et al. [109]	2015	Thermoplastic polyurethane	Sharklet micropattern by thermal embossing	Antiadhesion (bacteriocidal)	*S. aureus* (ATCC 6538) and *S. epidermidis* (ATCC 35984) (~10^7^ [static] or ~108 [dynamic] CFU/mL)	Colony counting, fluorescent microscopy (tryptic soy broth)	Reduction rate after 18 h colonization by 70% and 71% for *S. aureus* and *S. epidermidis*, respectively (< 1 log).	N A	N A
Tan et al. [110]	2024	Thermoplastic polyurethane	Micro‐nanostructure surface and superhydrophobic micro‐nanostructure surface grafted with hydroxyl silicone oil	Antiadhesion (bacteriocidal)	*E. coli* and *S. aureus* (10^8^ CFU/mL)	Fluorescent inverted microscope (lysogeny broth medium and M63 medium for *E. coli* and tryptic soy broth medium for *S. aureus*)	Rough surface reduced bacteria attachment by 66.7% reduction and 23.3% reduction for *E. coli* and *S. aureus*, respectively (< 1 log); the superhydrophobic surface with silicone oil reduced bacteria attachment by 23.3% reduction (< 1 log) and 97.9% (1.7 log) for *E. coli* and *S. aureus*, respectively.	N A	N A
Gao et al. [111]	2022	Polyurethane	Ordered hemisphere patterns	Antiadhesion (bacteriocidal)	*S. aureus* (CICC 10384) (dynamic retention assessment: 10^6^ CFU/mL, in situ real‐time investigation: 10^5^ CFU/mL)	FESEM, Nikon Eclipse Ti‐S inverted microscope (tryptic soy broth)	The most notable result was obtained for the 2‐*μ*m patterned PU film at 4.0 h: 37.5% bacterial reduction (< 1 log).	N A	N A
Mrad et al. [112]	2010	Polyurethane (pellethane 2363‐80AE)	Grafted oxygen and nitrogen species onto the surface with a low‐temperature plasma (smoother surface)	Antiadhesion /	*S. aureus* (CIP 4.8) (5 *x* 10^8^ *C* *F* *U*/*m* *L*)	Colony counting (sodium chloride–peptone water ~sodium chloride 8.5 g/L; bactopeptone 1 g/L per liter distilled water.)	Let the bacteria attach better by up to 0.57 log for 10 and 30 min.	N A	N A
Restivoet al. [113]	2024	Thermoplastic polyurethane	Topography modified by brush and bar coater deposition techniques	Antiadhesion (bacteriostatic)	*E. coli* (ATCC 25922) and *S. aureus* (ATCC 25923) (10^7^ CFU/sample)	Turbidity measurements (Luria–Bertani broth [*E. coli*], tryptic soy broth [*S. aureus*])	Nonadhesive properties of TPU against *E. coli* are independent of the type of deposition method. *S. aureus* was viable on film (~50%), brush (~70%), and bar coater (~25%) (< 1 log).	No	N A

**Table 10:** Other antiadhesive compounds—Other natural compounds. Abbreviations used: N A = not assessed; T = tested;  ^“^/^”^ = information not provided.

Author	Year	Material	Modification	AM category	Microorganism	Testing method/broth	Inhibition remark/inoculum	Cytotoxicity	In vivo
PEG									
Cao et al. [114]	2023	Polyaddition of an oligomer with isophorone diisocyanate and 1,4‐butanediol units generates a biodegradable polyurethane.	Dihydroxyl‐terminated poly[(ethylene oxide)‐co‐(ethylene carbonate)] (PEOC), polyethylene glycol (PEG), and polypropylene glycol (PPG) (backbone).	Antiadhesion (bacteriocidal)	*E. coli* and *S. aureus* (10^9^ cells/mL)	Microscope (Luria–Bertani broth for *E. coli* and tryptic soy broth for *S. aureus*)	PEOC‐PU: 2.3‐log reduction, PEG‐PU: 2.3‐log reduction, PPG‐PU: 0.5‐log reduction.	N A	N A
Meng et al. [115]	2018	Polyurethane.	Propargylic mPEG was grafted on the prepared azido‐containing polyurethane films via click chemistry (pendant).	Antiadhesion (bacteriocidal)	*S. aureus* and *E. coli* (3 *x* 10^5^ *C* *F* *U*/*m* *L*)	Colony counting, live/dead bacterial viability assay (tryptic soy broth)	Reduction of the adhered bacteria for approximately 75–97% (0.6 to 1.5 log).	N A	N A
Cao et al.	2024	Polyurethane.	Polyethylene glycol, QACs, and sulfobetaine (pendant).	Antiadhesion (bacteriocidal)	*S. aureus*, *E. coli*, and *P. aeruginosa* (5 *x* 10^7^ *c* *e* *l* *l* *s*/*m* *L*)	Inverted fluorescence microscope (Eclipse Ni–U, Nikon) (Luria–Bertani broth)	Exhibited an impressive reduction in bacterial adhesion, of over 96% (1.4‐log).	N A	N A
Li et al. [116]	2024	Hydrophilic PU elastomer developed by crosslinking the hydrophobic hard‐segment chains containing diselenide with diaminopyrimidine‐capped polyethylene glycol.	2,2 ^′^‐diselenodiethanol with diaminopyrimidine‐capped PEG and ethylene glycol (backbone).	Contact killing and antiadhesion (bacteriocidal)	*S. aureus* (10^6^ CFU/mL)	Colony counting, SEM, Kirby–Bauer test, turbidity measurements (tryptic soy broth)	17% bacterial reduction (< 1 log) and no zone of inhibition.	No	T (histocompatibility)
Fluor									
Qiao et al. [117]	2019	Polyurethane.	Fluorocarbon chains (pendant).	Antiadhesion (bacteriostatic)	*S. aureus* (colony assay: 10^5^ CFU/mL Baclight bacterial viability assay: 10^6^ CFU/mL)	Colony counting live/dead staining (tryptic soy broth)	Over 90% bacterial reduction (> 1 log).	N A	N A
Other compounds									
Chen et al. [118]	2020	Polyurethane acrylate.	Polyborneol acrylate/borneol groups (pendant).	Antiadhesion (bacteriostatic)	*E. coli* (5 *μ*L of 10^7^ CFU/mL)	Colony counting (/)	Biggest reduction for 54 wt. % Endo‐L‐Borneol acrylate: (100%) (4 log).	No	N A
Wu et al. [119]	2018	Waterborne polyurethane.	Isobornyl acrylate (pendant).	Antiadhesion (bacteriostatic)	*E. coli* and *S. aureus* (10^7^ CFU/mL)	Colony counting, turbidity measurements (beef‐protein liquid medium)	Inhibition rates for up to 89.3% and 80.4% for *E. coli* and *S. aureus*, respectively (> 1 log).	N A	N A
Villa et al. [120]	2009	Polyurethane.	N‐vanillylnonanamide (blend).	Antiadhesion (/)	*P. stutzeri* and *B. cereus* (10^6^ cells/mL)	Colony counting, ImageJ (plate count broth)	The coating was not able to reduce bacterial adhesion across all surfaces, neither for *P. stutzeri* nor for the *B. cereus* group (> 1 log).	N A	N A

**Table 11:** Extracted information—Dual compound: Halogen‐containing compounds. Abbreviations used: N A = not assessed; T = tested;  ^“^/^”^ = information not provided.

Author	Year	Material	Modification	AM category	Microorganism	Testing method/broth	Inhibition remark/inoculum	Cytotoxicity	In vivo
Lin et al. [36]	2015	Polyurethane	Fluorinated N‐halamine, 1‐chloro‐3‐1H,1H,2H,2H‐perflurooctyl‐5,5‐dimetylhydantoin, and its unfluorinated counterpart, 1‐chloro‐3‐octyl‐5,5‐dimethylhydantoin (blend)	Contact killing and antiadhesion (bacteriocidal)	*S. epidermidis* (ATCC 35984) and *A. calcoaceticus* (ATCC 31926) (10^8^–10^9^ CFU/mL)	Colony counting and SEM (nutrient broth solution)	At higher than 3% Cl‐ODMH content, no recoverable bacteria could be isolated (8‐ to 9‐log reduction); but even with 5% of Cl‐FODMH, *A. calcoaceticus* were recovered from the film (5 log for Cl‐ODMH and 3 log for Cl‐FODMH after 15 min).	N A	N A
Li et al. [121]	2018	Polyurethane	N‐chloramine and quaternary ammonium (pendant)	Contact killing (bacteriocidal)	*E. coli* (ATCC 25922) and *S. aureus* (ATCC 25923) (10^6^–10^7^ CFU/mL)	Colony counting (/)	*E. coli* 60.4% reduction and *S. aureus* 72.1% reduction (< 1 log).	N A	N A
Rogalsky et al. [122]	2021	Polyurethane LaripurLPR9020	Polymeric biocide polyhexamethylene guanidine hydrochloride (blend)	Contact killing and elution (bacteriocidal)	*E. coli* strain (GM 2163) and *B. subtilis* (strain 168) (1.5∙10^8^ CFU/mL for Kirby–Bauer) and (100 *μ*L of 1.5∙10^5^ CFU/mL for colony counting)	Kirby–Bauer test, colony counting (Luria–Bertani broth)	Total inhibition was detected for PU/PHMG‐Cl (2%) (4 log), a clear zone of inhibition was detected for PU/PHMG‐Cl (3%) sample, and this zone significantly increased for PU/PHMG‐Cl (4%).	N A	N A

**Table 12:** Extracted information—Dual compound: Quaternary compounds. Abbreviations used: N A = not assessed; T = tested;  ^“^/^”^ = information not provided.

Author	Year	Material	Modification	AM category	Microorganism	Testing method/broth	Inhibition remark/inoculum	Cytotoxicity	In vivo	
Xie et al. [123]	2022	Thermoplastic polyurethane.	Quaternized *β*‐chitin derivative‐ and quaternized chitosan derivative‐based (pendant)	Contact killing and antiadhesion (bacteriocidal)	*E. coli*, *S. aureus*, and MRSA (/)	SEM and colony counting (Luria–Bertani broth)	Quaternized chitosan: highest killing efficacies of 93% (< 1.2 log) for *E. coli*, 97% (1.5 log) for *S. aureus*, and 98% (1.7 log) for MRSA, respectively. —chitin: *E. coli* 57%, *S. aureus* 66%, MRSA 63% (< 1 log).	No	Subcutaneous implantation in mice	*S. aureus*, SEM and colony counting with a 99.87% reduction (up to 3 log)
Chen et al. [124]	2018	Fibrous polyurethane membrane.	Quaternary ammonium chitooligosaccharide immobilized via an intermediate layer of polydopamine (pendant)	Contact killing and antiadhesion (bacteriocidal)	*E. coli* and *S. aureus* (/)	Turbidity measurement, colony counting (Luria–Bertani medium)	The highest antibacterial efficacy up to 75.3% and 70.3% (< 1 log) was obtained for 10G‐C‐ D‐PU fibrous membrane after being in contact with *E. coli* and *S. aureus*, respectively, for 24 h.	No	N A	
Huang et al. [125].	2024	Polydopamine/polyethylenimine–modified polyurethane.	Polymerization of imidazolium salt chitosan, 2‐methacryloyloxyethyl phosphorylcholine, and acrylic acid (pendant)	Contact killing and antiadhesion (bacteriocidal)	*E. coli* (ATCC 25922) and *S. aureus* (CMCC(B) 26003) (1.0 × 10^8^ *C* *F* *U*/*m* *L*)	Colony counting, and live/dead bacterial staining assay (phosphate buffer saline solution)	Antibacterial rates of 99.94% (3.2 log) and 99.82% (2.7 log) against *E. coli* and *S. aureus.*	No	N A	
Kurt et al. [126]	2007	Polyurethane.	Alkylammonium (QAC) and either trifluoroethoxy or PEGylated side chain (pendant)	Contact killing (bacteriocidal)	*P. aeruginosa*, *E. coli* and *S. aureus* (10^7^ CFU/mL)	Colony counting, Kirby–Bauer test /	100% kill (3.6‐ to 4.4‐log reduction), no ZOI.	N A	N A	
Pinar Kurt and Kenneth [127]	2008	Copolyoxetane soft block polyurethanes.	Either fluorous or PEG‐like side chains with quaternary ammonium compounds (pendant)	Contact killin and antiadhesion (bacteriocidal)	*P. aeruginosa*, *E. coli*, and *S. aureus* (/)	Colony counting (/)	Affected 100% killing of sprayed‐on *P. aeruginosa*, *E. coli*, and *S. aureus*, resulting in a 3.6–4.4‐log reduction in 30 min.	N A	N A	
Wang et al. [128]	2019	Polyurethane.	Quaternary ammonium and PEG‐like side chains (pendant)	Contact killing (bacteriocidal)	HfrH *E. coli* strain 43 and *S. epidermidis* RP62A methicillin‐resistant strain 4 (colony counting: [1 − 3] × 10^5^ *C* *F* *U*/*m* *L*, Kirby–Bauer test: 1.1 × 10^5^ *C* *F* *U*/*m* *L*)	ASTM E2149‐01/colony counting, Kirby–Bauer test (M9 medium)	An average 3.7‐log reduction was found against *S. epidermidis*, whereas a > 4 log‐reduction was achieved against *E. coli* absence of a ZOI.	No	N A	
Zhang et al. [129].	2018	Cross‐linked waterborne polyurethane with long‐term stability developed from.	PEG, polyoxytetramethylene glycol, isophorone diisocyanate, L‐lysine, and its derivative diamine consisting of gemini quaternary ammonium salt, using ethylene glycol diglycidyl ether as a cross‐linker; QAS (pendant) and PEG (backbone and terminal)	Contact killing and antiadhesion (bacteriocidal)	*S. aureus* and *E. coli* (1 × 10^6^ *C* *F* *U*/*m* *L*)	Turbidity measurements, colony counting (nutrient broth)	More than 99.99% (> 4 log) bacteria in the local culture were killed in the surrounding environment; no zone of inhibition.	No	N A	
He et al. [130]	2016	Waterborne polyurethanes.	Sublayer: PEG (backbone), upper layer: gemini quaternary ammonium salt (GQAS) (pendant)	Contact killing and antiadhesion (bacteriocidal and bacteriostatic)	*E. coli* (ATCC 25922) and *S. aureus* (ATCC 6538) (10^7^ CFU/mL)	Colony counting, SEM (nutrient broth)	99.99% (4 log) killing efficiency against both Gram‐positive and Gram‐negative bacteria; no zone of inhibition.	N A	T (biocompatibility)	
Aydin et al. [131].	2018	Polyurethane.	Quaternary ammonium salt diol, and PEG (backbone)	Contact killing (bacteriocidal)	*S. aureus* and *E. coli* (10^4^ CFU)	ASTM 2149 method/colony counting (/)	95% (1.3 log) bacterial reduction for *S. aureus* and 38% (< 1 log) for *E. coli.*	N A	N A	
Coneskiet al. [132].	2012	Polyurethane	Hydroxyl‐terminated macrodiols and tethered quaternary ammonium biocides/poly(ethylene glycol)‐containing urethanes (backbone)	Contact killing and antiadhesion (bacteriocidal)	Antimicrobial test: *S. aureus* and *E. coli* (1 × 10^9^ *C* *F* *U*/*m* *L*) bioadhesion test: *P. aeruginosa* (1 × 10^9^ *C* *F* *U*/*m* *L*)	Colony counting, turbidity measurement (/)	90% (1 log) increase in biocidal activity compared with control materials, while reducing the ability for microbes to adhere to the surface by an additional 60% (< 1 log).	N A	N A	
Du et al. [133]	2018	Cationic waterborne polyurethane.	Guanidinoacetic acid is linked to polyurethane molecules to form a QAS with guanidine (pendant).	Contact killing, elution and antiadhesion (bacteriocidal)	*E. coli* and *B. subtilis* (1 *x* 10^6^ *c* *e* *l* *l* *s*/*m* *L*)	Colony counting, turbidity measurements (/)	Antibacterial efficiency *E. coli* (92.90%, 1.1 log) and *B. subtilis* (98.20%, 1.7 log).	N A	N A	
Zheng et al. [134].	2024	Plant oil–based waterborne polyurethane	Quaternary ammonium groups and mPEG side chains (pendant).	Contact killing (bacteriocidal)	*S. aureus* and *E. coli* (10^6−7^ CFU/mL)	Colony counting (lysogeny broth nutrient broth)	Antibacterial rate against *S. aureus* was 91.8% (1.1 log), whereas the rate against *E. coli* was 92.3% (1.1 log).	N A	N A	
Guo et al. [135]	2024	Waterborne polyurethane.	Quaternary ammonium salt and hydrophilic components like PEG (backbone)	Contact killing (bacteriocidal)	*E. coli* and *S. aureus* (/)	Colony counting (/)	Up to 82% bacterial reduction (< 1 log).	No	T (wound closure)	
Gupta et al. [136]	2025	Water‐dispersible polyurethane.	Quaternary ammonium moieties, PPG, and PEG (backbone)	Contact killing and elution (bacteriocidal)	*E. coli* and *S. aureus* (/)	Kirby–Bauer test (/)	Zones of inhibition measuring 7 and 8 mm (*E. coli* and *S. aureus*).	No	N A	
Sharma et al. [137]	2021	Polydimethyl siloxane–modified polyurethanes.	Covalently modified with quaternized ammonium (pendant), pyridinium, and phosphonium compounds	Contact killing and antiadhesion (bacteriocidal)	*E. coli* (K12 MG 1655 wild type) and *P. mirabilis* (ATCC 51286, methicillin‐resistant *S. aureus* (MRSA, USA 300) (10^8^–10^9^ CFU/mL)	Colony counting, SEM (Luria broth)	Nearly 3‐log reduction.	No	N A	
Liu et al. [138]	2020	Thermoplastic polyurethane.	Amphiphilic carbonaceous particles, quaternized polyethyleneimine, and thermoplastic polyurethane were constructed by a carrier dispersion strategy (QACs as pendant in particles, particles blended in PU).	Contact killing and antiadhesion (bacteriocidal)	*S. aureus*, *E. coli*, *C. albicans*, and MRSA (4 × 10^6^ *C* *F* *U*/*m* *L*)	Turbidity measurement, and live/dead staining (/)	97% (1.5 log) bacteria of different species lose their viability after contacting with TPU/ACPs‐QPEI.	No	Implanted subcutaneously via the incision into the inner thigh tissue of mice	MRSA suspension (1 × 10^6^ *C* *F* *U*/*m* *L*), colony counting with over 90% antimicrobial efficacy (> 1 log)
Jiang et al. [139].	2024	Polyurethane (PU)–based coatings.	Quaternized ammonium–modified methyldiethanolamines (pendant) and a hydrophilic polymer, polyvinylpyrrolidone (blend)	Contact killing and antiadhesion and elution (bacteriocidal)	*S. aureus* and *E. coli* (/)	Kirby–Bauer test, SEM /	Antibacterial efficiency of 99.9% (3 log), no inhibition zone under 3% QMDEA‐C10.	No	Subcutaneously implanted into mice: An infected animal model	
Shi et al. [140].	2024	Polyurethane	Bisfuran‐based polyamide polymers with various functional groups, including allyl quaternary ammonium cations, decyl quaternary ammonium cations, and sulfonate betaine (pendant).	Contact killing and antiadhesion (bacteriocidal)	*E. coli* and *S. aureus* (10^8^ CFU/mL)	Colony counting, SEM, and live/dead staining /	Even after seven cycles of use, the coatings maintained the ability to kill ~80% (< 1 log) and > 99% (> 2 log) of *E. coli* and *S. aureus* bacteria, respectively.	No	N A	
Zhu et al. [141].	2025	Polyurethane.	[3‐(methacryloylamino)propyl] trimethylammonium chloride, zwitterionic sulfobetaine methacrylate, and quaternary ammonium copolymers grafted on PU (pendant)	Contact killing and antiadhesion (bacteriocidal)	*S. aureus*, *E. coli*, and *P. aeruginosa* (5 × 10^7^ *c* *e* *l* *l* *s*/*m* *L*)	Fluorescence microscope: live/dead staining and quantification with Image J software (Luria–Bertani broth)	Capacity to eliminate in excess of 99.0% (2 log) of bacteria following a 7‐day period of contact.	N A	N A	
Cao et al. [142]	2024	Polyurethane.	Polyethylene glycol, QACs, and sulfobetaine (pendant)	Antiadhesion (bacteriocidal)	*S. aureus*, *E. coli*, and *P. aeruginosa* (5 *x* 10^7^ *c* *e* *l* *l* *s*/*m* *L*)	Inverted fluorescence microscope (Eclipse Ni–U, Nikon) (Luria–Bertani broth)	Exhibited an impressive reduction in bacterial adhesion, with over 96% (1.4 log).	N A	N A	
Levana et al. [143].	2023	Thermoplastic polyurethane (TPU) embedded with bentonite and MTT clay containing QAS.	Quaternary ammonium salts in montmorillonite clay (blend)	Contact killing, elution and antiadhesion (bacteriocidal)	*S. aureus* (KCTC3881) and *E. coli* (ATCC25922‐GFP) (10^8^ CFU/mL)	Turbidity measurements, SEM (Miller′s Luria–Bertani broth)	85.34% and 82.74% reduction (< 1 log) in *E. coli* and *S. aureus* adhesion and killing efficiency.	No	N A	
Jia et al. [144, 145]	2020	Cationic waterborne polyurethane.	Quaternary ammonium salts and siloxane (pendant)	Contact killing and antiadhesion (bacteriocidal)	*E. coli* and *S. aureus* (10^8^ CFU/mL)	Kirby–Bauer test (/)	ZOI: 13.71 mm.	N A	N A	

**Table 13:** Extracted information—Dual compound: Guanidine. Abbreviations used: N A = not assessed; T = tested;  ^“^/^”^ = information not provided.

Author	Year	Material	Modification	AM category	Microorganism	Testing method/broth	Inhibition remark/inoculum	Cytotoxicity	In vivo
Liu et al. [146]	2021	Cationic waterborne polyurethane	Chitosan biguanide hydrochloride (backbone)	Contact killing (bacteriocidal)	*E. coli* and *S. aureus* (3 × 10^5^ *C* *F* *U*)	Colony counting (beef paste 0.3 g, peptone1 g, and NaCl 0.5 g were added to 100 mL of distilled water).	Antibacterial rate against *S. aureus* and *E. coli* reached 91% (1 log) and 85% (< 1 log), respectively.	N A	N A
Gharibi and Agarwal [147]	2021	Thermoplastic polyurethane	Polyguanidine and oxidized dextran (pendant)	Contact killing and antiadhesion (bacteriocidal)	Leachate extracts: *B. subtilis* antibacterial kinetics: *E. coli* (10^6^, 10^5^, 10^4^, and 10^3^ CFU/mL) antibiofilm Assay: *E. coli* (5 mL of ∼2 × 10^8^ *C* *F* *U*/*m* *L*)	Antibacterial activity on leachate extracts from films: turbidity measurements and colony counting antibacterial kinetics: colony counting antibiofilm assay: SEM nutrient broth.	Exhibited 100% killing efficiency (8 log) against both bacteria.	No	N A
Yuan et al. [148]	2021	Polyurethane	Polyhexamethylene guanidine/hyaluronic acid (pendant)	Contact killing and antiadhesion (bacteriocidal)	*E. coli* (DH5 alpha), *S. aureus* (ATCC 6538), and *P. aeruginosa* (ATCC 2785) 10^8^ CFU/mL	Colony counting, live/dead staining, lysogenic broth medium.	Inhibition rates of *E. coli*, *P. aeruginosa*, and *S. aureus* were 99.99% (4 log), 99.96% (3.4 log), and 99.99% (4 log), respectively.	No	N A

**Table 14:** Extracted information—Dual compound: Other synthetic compounds. Abbreviations used: N A = not assessed; T = tested;  ^“^/^”^ = information not provided.

Author	Year	Material	Modification	AM category	Microorganism	Testing method/broth	Inhibition remark/inoculum	Cytotoxicity	In vivo
Sharma et al. [149].	2022	Polydimethylsiloxane modified thermoplastic polyurethane blend	Grafted branched polyethyleneimine and poly(2‐ethyl‐2‐oxazoline) (layer‐by‐layer assembly route) (pendant).	Contact killing and antiadhesion (bacteriocidal)	*P. mirabilis* (ATCC 51286), methicillin‐resistant *S. aureus* (MRSA, USA 300), *P. mirabilis* (/)	Colony counting, resazurin reduction assay, live/dead staining (Luria–Bertani broth)	4‐fold log reduction in the planktonic growth.	No	N A
Liu et al. [150].	2023	Waterborne polyurethane	Dimethyloctadecyl[3‐(trimethoxysilyl) propyl] ammonium chloride, benzalkonium chloride, and octenidine dihydrochloride (pendant).	Contact killing (bacteriocidal)	*E. coli* and *S. aureus* (10^7^ CFU/mL)	Colony counting (nutritional broth)	More than 99% (2 log) bacterial reduction for both *E. coli* and *S. aureus*; remained more than 90% (1 log) after 7 days of immersion in buffer solution.	N A	N A
Chung et al. [151].	2019	Polyurethane	Tetracycline, as an attached functional group (pendant), and dimethylolpropionic acid and polyol were incorporated.	Contact killing and antiadhesion (bacteriocidal)	*S. aureus* (ATCC 6538) and *K. pneumoniae* (ATCC 4352) (/)	KS K 0693‐2016: Colony counting (/)	Bacterial reduction > 99.99998% of *S. aureus* (> 6.7 log); > 99.9999995% *K. pneumoniae* (> 8.3 log).	N A	N A
Gharibi et al. [152]	2015	Polyurethane/siloxane	Methoxysilane functional aniline tetramer moieties (pendant).	Contact killing (bacteriocidal)	*S. aureus* (ATCC 6538) and *P. aeruginosa* (ATCC 15449) bacteria (/)	Colony counting (/)	Almost 100% bacterial reduction against all strains (inoculum is not defined, so log reduction is unclear).	No	T (wound healing)
Wenet al. [153]	2021	Polyurethane	1,2,3‐triazole link and N‐vinylpyrrolidone (pendant)	Contact killing and antiadhesion (bacteriostatic)	*S. aureus*, *E. coli*, and *P. aeruginosa* (10^8^ CFU/mL)	Live/dead staining (tryptic soy broth)	Reduction in bacterial attachment with 26%–67%, 24%–61%, and 23%–57% (< 1 log) decrease to *S. aureus*, *E. coli*, and *P. aeruginosa*, respectively.	N A	N A

**Table 15:** Extracted information—Dual compound: Natural compounds. Abbreviations used: N A = not assessed; T = tested;  ^“^/^”^ = information not provided.

Author	Year	Material	Modification	AM category	Microorganism	Testing method/broth	Inhibition remark/inoculum	Cytotoxicity	In vivo
Karaet al. [154].	2015	Hexamethylene diisocyanate‐based polyurethanes	Immobilized chitosan and heparin (pendant).	Contact killing (bacteriocidal)	*E. coli* (ATCC 11229) and *P. aeruginosa* (ATCC 27853), *S. aureus* (ATCC 25923), and *S. epidermidis* (ATCC 12228) (10^7^–10^8^ CFU/mL)	Colony counting (nutrient broth)	A combination of chitosan‐heparin was found to be the most effective at > 3.5‐log reduction.	N A	N A
Karaet al. [155].	2016	Polyurethane	Grafted chitosan and heparin (pendant)	Contact killing and antiadhesion (bacteriocidal).	*P. aeruginosa* (ATCC 27853), *E. coli* (ATCC 11229), *S. aureus* (ATCC 25923), and *S. epidermidis* (ATCC 12228) (100 *μ*L of 10^7^–10^8^ CFU/mL)	Colony counting and SEM (nutrient broth)	Samples killed all bacteria after 24 h inhibition. (6–7 log reduction).	No	N A
Li et al. [156].	2024	Nonsoluble antibacterial polyurethane	Grafted chitosan azide and heparin azide (pendant).	Contact killing and antiadhesion (bacteriocidal)	*E. coli* (ATCC 25 922) and *S. aureus* (ATSS 6538) (1 × 10^5^ *C* *F* *U*/*m* *L*)	Colony counting nutrient broth (nutrient broth)	The highest antibacterial rate was 92.07% (1.1 log).	No	N A
Luo et al. [157].	2017	Polyurethane	Chitooligosaccharide was modified onto the surface of PU membrane based on the self‐polymerization of dopamine (pendant).	Contact killing (bacteriocidal)	*E. coli* (ATCC 8739) and *S. aureus* (ATCC6538P) (/)	Colony counting (Luria–Bertani broth)	*E. coli* and *S. aureus*: 35.44% and 48.78% antibacterial efficacy (0.48 log).	No	N A
Hu et al. [158].	2023	Polyurethane adhesive blend coated in the blend on coelectrospinning cellulose acetate/thermoplastic polyurethanes composite membrane	Photosensitizer phthaloyl phthalic acid/antimicrobial agent caffeic acid integrated into a polyurethane binder blend.	Contact killing and Elution (bacteriocidal)	*E. coli* and *S. aureus* (1 × 10^5^ *C* *F* *U*/*m* *L*)	Colony counting, SEM (phosphate buffer saline (pH 7.4))	The bactericidal rates of *S. aureus* and *E. coli* reached 99.2% (2.1 log) and 93.4% (1.2 log), respectively.	N A	N A

**Table 16:** Extracted information—Dual compound: Antiadhesive compounds. Abbreviations used: N A = not assessed; T = tested;  ^“^/^”^ = information not provided.

Author	Year	Material	Modification	AM category	Microorganism	Testing method/broth	Inhibition remark/inoculum	Cytotoxicity	In vivo
Kim et al. [159]	2024	Polyurethane acrylate	Films with variously sized holes (ranging from 0.3 to 4 mm) and with the zwitterionic polymer 2‐methacryloyloxyethyl phosphorylcholine coated onto the nanohole pattern surface	Antiadhesion (bacteriocidal)	*S. aureus* (ATCC 25923) (10^7^ CFU/mL)	Colony counting, live/dead staining, fluorescence, and SEM (Luria broth)	Inhibited the colonization of *S. aureus* (18 h; 82%, 7 days; 83%, and 14 days; 68% antibacterial rate) (> 1 log)	N A	N A
Xu et al. [160].	2018	Polyurethane	Submicron pillars and fluorocarbon (poly[bis(octafluoropentoxy) phosphazene] blended with the polyurethane).	Antiadhesion (bacteriostatic)	*S. epidermidis* RP62A (ATCC 35984) (1 *x* 10^8^ *C* *F* *U*/*m* *L*)	Fluorescence optical microscopy (tryptic soy broth)	75% reduction of adhesion on film surfaces (> 1 log).	N A	N A
Xu et al. [161]	2017	Polyurethane	Ordered pillar topographies at the top surface and a S‐nitroso‐N‐acetylpenicillamine (NO donor) doped sublayer in the middle, via a soft lithography two‐stage replication process.	Elution (bacteriocidal) and antiadhesion (bacteriostatic)	*S. epidermidis* RP62A (ATCC 35984) (NO‐release: 1 × 10^6^ CFU/mL bacterial adhesion: 1 × 10^8^ *C* *F* *U*/*m* *L*, biofilm formation: 1 × 10^8^ *C* *F* *U*/*m* *L*)	Turbidity and SEM (tryptic soy broth)	88% reduction rate (> 1 log)	N A	N A
Walter et al. [162]	2014	Polyurethane	Urethane moieties, polyethylene glycol, and polypropylene glycol (no specific AM groups).	Contact killing (bacteriocidal)	*S. aureus* (ATCC 6538) on *E. coli* (ATCC 8739) (10^8^ CFU/mL)	Kirby–Bauer test (nutrient agar medium)	PU‐PEG 100%: *S. aureus*: 15 mm, *E. coli*: 14 mm, PU‐PPG 100%: *S. aureus*: 15 mm, *E. coli*: 16 mm.	N A	N A
Ajit Walter et al. [163].	2015	Poly(urea–urethane–imide) hybrid	Imide moiety and polydimethylsiloxane and polyhedral oligomeric silsesquioxane (backbone).	Contact killing, antiadhesion, and elution (bacteriocidal)	*S. aureus* (ATCC 6538) and *E. coli* (ATCC 8739) (25 mL of 10^8^ CFU/mL)	Kirby–Bauer test, live/dead backlights bacterial viability and SEM (nutrient agar broth)	ZOI: 13 mm for *S. aureus* and 15 mm for *E. coli* reduction of bacteria attachment: ~99% (~2‐log reduction) for *S. aureus* and~96% (~1‐log reduction) for *E. coli.*	No	N A
Rossi de Aguilar et al. [164].	2019	Poly(urethanic) hybrid materials based on polydimethylsiloxane	Polyoxometalate (phosphotungstic acid–PWA) (a special composite with a caged metal)	Antiadhesion (bacteriostatic).	*E. coli* and *L. casei* (50 *μ*L of 1*x*10^6^ *C* *F* *U*/*m* *L*)	Turbidity measurements (Luria–Bertani broth (*E. coli*) and De Man–Rogosa–Sharpe broth [*L. casei*])	The adhesion of Gram‐positive and Gram‐negative bacteria is suppressed in 4 h of contact (4‐log suppression).	No	N A

**Table 17:** Extracted information—In vivo studies. Abbreviations used: N A = not assessed; T = tested;  ^“^/^”^ = information not provided.

Author	Year	Material	Modification	AM category	Microorganism	Testing method/broth	Inhibition remark/inoculum	Cytotoxicity	In vivo	
Peng et al. [70]	2023	Zwitterionic polyurethane polymers were dipcoated onto a thermoplastic polyurethane substrate.	Quaternary amine groups (backbone)	Contact killing (bacteriocidal)	*E. coli* and *S. aureus* (/)	Live/dead staining and colony counting (/)	Bactericidal ratios of the coatings against *E. coli* and *S. aureus* reached 99.9% (3 log).	No	Reoperative infection model in the subcutaneous tissue of SD rats.	*S. aureus*, colony counting with a 2–3 log reduction
Xie et al. [123].	2022	Thermoplastic polyurethane.	Quaternized *β*‐chitin derivative‐ and quaternized chitosan derivative–based (pendant)	Contact killing and antiadhesion (bacteriocidal)	*E. coli*, *S. aureus*, and MRSA (/)	SEM and colony counting (Luria–Bertani broth)	Quaternized chitosan: highest killing efficacies of 93% (< 1.2 log) for *E. coli*, 97% (1.5 log) for *S. aureus*, and 98% (1.7 log) for MRSA, respectively. —chitin: killing efficacies of *E. coli* is 57%, *S. aureus* is 66%, and MRSA 63% (< 1 log).	No	Subcutaneous implantation in mice	*S. aureus*, SEM and colony counting with a 99.87% reduction (up to 3 log)
Liu et al. [138]	2020	Thermoplastic polyurethane.	Amphiphilic carbonaceous particles, quaternized polyethyleneimine, and thermoplastic polyurethane were constructed by a carrier dispersion strategy (QACs as pendant in particles, particles blended in PU)	Contact killing and antiadhesion (bacteriocidal)	*S. aureus*, *E. coli*, *C. albicans*, and MRSA (4 × 10^6^ *C* *F* *U*/*m* *L*)	Turbidity measurement, and live/dead staining (/)	97% (1.5 log) bacteria of different species lose their viability after contacting with TPU/ACPs‐QPEI.	No	Implanted subcutaneously via the incision into the inner thigh tissue of mice	MRSA suspension (1 × 10^6^ *C* *F* *U*/*m* *L*), Colony counting with over 90% antimicrobial efficacy (> 1 log)
Jiang et al. [139]	2024	Polyurethane‐based coatings.	Quaternized ammonium‐modified methyldiethanolamines (pendant) and a hydrophilic polymer, polyvinylpyrrolidone (blend)	Contact killing and antiadhesion and elution (Bacteriocidal)	*S. aureus* and *E. coli* (/)	Kirby–Bauer test, SEM /	Antibacterial efficiency of 99.9% (3 log), no inhibition zone under 3% QMDEA‐C10.	No	Subcutaneously implanted into mice: an infected animal model	/, *S. aureus* with 87.48% and 83.35% (> 1 log) bacterial reduction
Dhyani et al. [101]	2024	Polyurethane.	Two different EO components: alpha‐terpineol and cinnamaldehyde (terminal)	Contact killing and elution (bacteriocidal)	*E. coli*, methicillin‐resistant *S. aureus*, and *P. aeruginosa* (~10^6^ CFU/mL)	Colony counting (tryptic soy broth)	Up to 6‐log bacterial reduction.	N A	In vivo full‐thickness porcine burn model	*S. aureus* 10^7^ CFU/mL with > 5‐log reduction on Days 2 and 3
Lu et al. [98]	2021	Thermoplastic polyurethane.	Thermoplastic polyurethane surface modification of peptide polymer using plasma surface activation and substitution reaction between thiol and bromide groups (pendant)	Contact killing (bacteriocidal)	Methicillin‐resistant *S. aureus* (USA 300), *S. haemolyticus* (R01), *E. coli* (JM109), and *P. aeruginosa* (9027) (5 × 10^5^ *C* *F* *U*/*m* *L*)	Colony counting, leaching assay (fluorescence), SEM (/)	Showing killing efficacy of 99.9% (3 log) against *E. coli*, 97.5% (1.6 log) against MRSA, 99.9% (3 log) against *S. haemolyticus*, and 91.2% (1.1 log) against *P. aeruginosa*, respectively, no leaching.	No	Subcutaneous implantation infectious model with (SD) rats	MRSA (5 × 10^5^ *C* *F* *U*/*m* *l*), colony counting with a 1.27‐log reduction

There was substantial variability in the reporting practices among the studies included in this review. To facilitate standardization and enable meaningful comparisons, all antimicrobial efficacy results are reported on a logarithmic scale, except when the reduction is less than 1 log. In such cases, the data are presented as a percentage reduction to make comparing low bacterial reductions easier; however, bacterial reductions of this magnitude are generally not considered clinically relevant. When a study did not explicitly state the antimicrobial reduction achieved, this value was estimated based on graphical representations or other available data.

Compounds were classified as bacteriostatic or bactericidal, and further categorized as antiadhesive and/or contact‐killing. In cases where a compound also exhibited elution alongside antiadhesive and/or contact‐killing activity, this characteristic was specifically documented. When such classifications were provided in the original studies, they were adopted in this review. In the absence of such classifications, designations were made based on the best fit according to the information provided. In the absence of a standardized method for distinguishing between bacteriostatic and bactericidal activity, and given the limitations of the available data, a simplified classification approach was adopted: Compounds showing a reduction in bacteria count over time were defined as bactericidal, whereas those maintaining a stable bacterial load were considered bacteriostatic. It was also assessed if the antimicrobial compound was located in the backbone, pendant, or terminal group of the polyurethane or if it was blended into the polyurethane. All information is provided in the tables summarizing the results of each category.

## 3. Results

The database search located 2082 studies. The number of studies was reduced to 980 after excluding all duplicates. After screening the titles and abstracts of all studies, 564 studies were excluded. Following full‐text screening, an additional 282 studies were excluded, leaving 134 studies included in this review (Figure 1).

The studies were categorized by the antimicrobial compound or strategy that was incorporated into the polyurethanes. All compounds that work through a contact‐killing antimicrobial mechanism were further divided into synthetic or natural. Afterward, strategies and compounds used to modify polyurethanes to prevent bacterial attachment were included in a separate category. Lastly, all studies with polyurethanes modified with two different strategies or compounds have been included under the category of polyurethanes modified with several compounds.

An overview of the number of antimicrobial studies investigating both polyurethane matrices containing a single antimicrobial compound as well as multiple compounds is provided in Figure 2.

Figure 2Schematic overview of the type of antimicrobial compound studied in polyurethane matrices as investigated in this systematic review. (a) Overview of studies investigating a single antimicrobial compound in polyurethane matrices. (b) Overview of studies investigating multiple antimicrobial compounds in polyurethane matrices.

**Figure:**
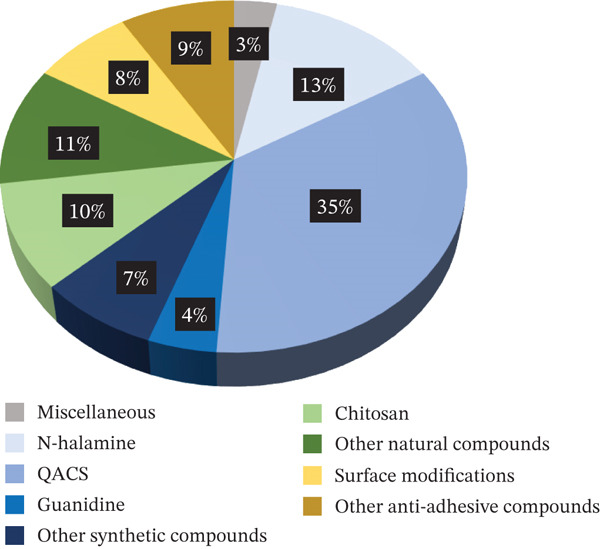
(a)

**Figure:**
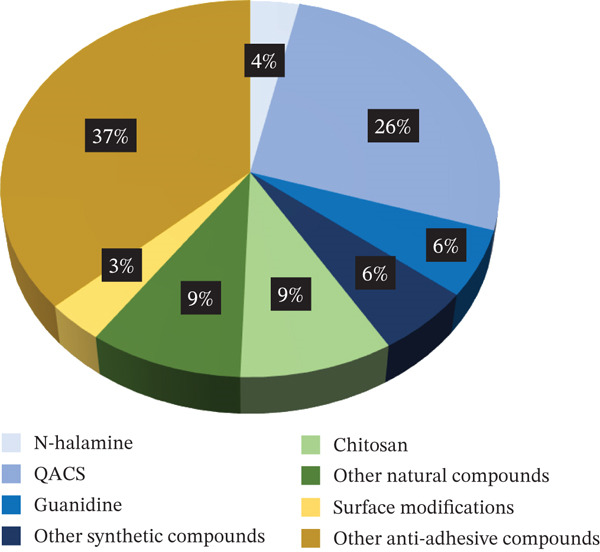
(b)

(a) Studies on PU containing a single antimicrobial compound

(b) Studies on PU containing several antimicrobial compounds

### 3.1. Miscellaneous Polyurethanes

There appears to be a difference in opinion regarding the antimicrobial properties of pure polyurethane. Although most studies claim that polyurethane is not antimicrobial without modification [165–167] there are two studies by Walter et al. [162, 163] In this review, it is claimed that urethane moieties specifically possess biocidal abilities. Since the polyurethane membrane they investigated also contained either polypropylene glycol and poly(ethylene glycol) (PEG) [162] or silsesquioxane [163], it is not clear how much of its antimicrobial properties are based on the urethane moieties, and this will be further discussed in the dual‐compound section.

In the literature search for this review, three studies were found that discuss antimicrobial polyurethanes but do not specify whether the polyurethanes were modified to be antimicrobial or explain their mode of action. It is unclear whether the antimicrobial properties of these polyurethanes are due to a biocidal ability of the urethane moieties or if the compounds used to synthesize the polyurethanes made them antimicrobial. None of the studies exhibited a high bacterial reduction. Uscategui et al. [30] reported the highest bacterial reduction (77%) with a polyurethane synthesized from polycaprolactone. In 2019, Uscategui et al. [31] also reported a 7.5% bacterial inhibition for a polyurethane made from castor oil with long aliphatic side segments. Finally, Kultys and Puszka [32] synthesized poly(thiourethane‐urethane)s with a 64.4% bacterial reduction according to turbidity measurements. Table 1 summarizes the principle outcomes from these studies.

### 3.2. Contact‐Killing Modifications

Contact‐killing surfaces are capable of killing microbes by binding biocides to the surface [18]. The most well‐known contact‐killing biocides include synthetic compounds like quaternary ammonium compounds (QACs), N‐halamines, and guanidines [18], and natural compounds like chitosan [168]. This chapter focuses exclusively on polyurethanes containing a single contact‐killing compound. Polyurethanes incorporating multiple compounds are addressed separately in the category of polyurethanes modified with several compounds.

#### 3.2.1. Synthetic Organic Compounds

The number of synthetic antimicrobial compounds in research has been growing recently [169]. Synthetic compounds are categorized by their chemical structure [170], for example, quaternized compounds or compounds containing halogens like N‐halamines [169].

##### 3.2.1.1. Halogen‐Containing Compounds

**Figure 3:**
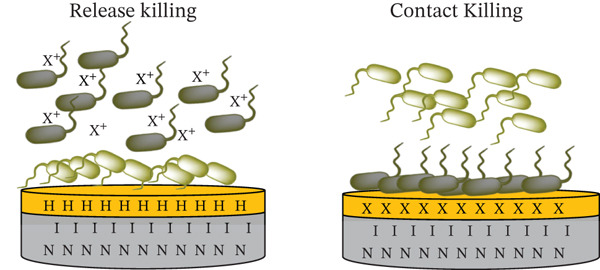
Schematic representation demonstrates the differences between release‐based (eluting) antimicrobial systems and surface‐based (noneluting or contact‐killing) antimicrobial systems (made with Biorender).

N‐halamines are potent biocides with one or several nitrogen atoms covalently bonded to oxidative halogens [33, 171]. The antimicrobial mechanism is primarily thought to be either contact killing, release killing, or a combination of both [43]. The contact‐killing mechanism involves the direct transfer of the positively charged halogens to specific cell receptors without releasing them into the solution, which can inhibit or destroy vital cell functions [33, 171, 172]. Meanwhile, the release‐killing mechanism causes the covalent bond between the nitrogen and halogen ion to break, allowing the ion to dissolve into the solution (Figure 3) [172]. Studies report that more stable halogen bonds tend to work primarily by a contact‐killing mechanism, whereas less stable bonds result in a more release‐based biocidal mechanism [172, 173]. A big advantage of this compound is that the biocidal mechanism can be regenerated by adding free halogen ions, for example, bleach [41].

A zone of inhibition test is often utilized to investigate if a material releases biocides. In two of the studies reviewed of polyurethanes containing N‐halamines, no zone of inhibition was observed, and bacteria could be fully eradicated after 5 min (6‐ to 7‐log reduction) [33] or after 120 min (4‐ to 5‐log reduction) [34]. In contrast, a study of a polymer network containing N‐halamine reported a small but distinct clear zone of inhibition and the eradication of all bacteria after 4 h (5‐ to 6‐log reduction) [35]. The other N‐halamine/PU studies did not investigate how much of the reported bacterial reduction is due to a release or contact‐killing mechanism. The studies reported that the bacterial load was completely reduced after 15 min (8‐ to 9‐log reduction) [36], after 2 h (6‐log reduction) [37], and (4‐log reduction) [38] or after 30 min (6‐ to 8‐log reduction) [39]; they reported a bacterial reduction of about 4.7 log after 3 h [40], of up to 5.88 log after 2 h, [41], of up to 4.38 log after 30 min [42], or > 1.3‐log after 5 min [43].

Polyurethanes have also been modified with other halogen‐containing compounds. Raut et al. [44] investigated polyurethane–polyvinylpyrrolidone iodine films and reported up to 2‐log reduction in bacterial adhesion. The primary outcomes from the studies discussed in this paragraph are summarized in Table 2.

##### 3.2.1.2. Quaternary Compounds

QACs constitute a large category of antimicrobial compounds. There is no consensus in the literature about their antimicrobial mechanism [69]. It is theorized that, first, the bacteria adsorb to the positively charged QACs through their negatively charged cell walls, mediated by electrostatic forces [48, 53, 64]. Then, the moieties diffuse into the cell wall and disrupt the bacterial membrane, releasing the intracellular material and killing the bacteria [48, 53, 69]. Another theory proposes that the electrostatic interaction between the QACs and the bacterial membrane leads to the formation of an impermeable layer around the cell, which stops nutrient permeation [124, 174]. Unfortunately, because of the widespread use of QACs, there is an increasing number of bacteria developing resistance to them. This makes the search for new antimicrobial QACs progressively important [54].

The antimicrobial reduction in the QAC studies reviewed, which had no zone of inhibition, was up to 5 log [45–47], 100% (4‐log reduction) [48, 49] and between over 99.9% (3‐log reduction) [50, 51] and 25% [50, 52–56].

In contrast, Hua and Odelius [57] investigated a polyhydroxyurethane film containing QACs and iodide counterions as a dual killing mechanism (diffusion and contact‐active killing). They reported a bacterial reduction of 3 log and clear zones of inhibition. Wang et al. [58] investigated polyurethane incorporating QAS‐spandex fibers. A zone of inhibition of up to 26.3 mm and antibacterial ratios of up to 89.7% was observed. Rabiee et al. [59] made bioadhesives functionalized with methacrylate and quaternary ammonium (QA) groups. Depending on the concentration, they reported a zone of inhibition and up to 66% bacterial reduction. The remaining studies on polyurethanes containing QACs did not explore whether the bactericidal mechanism is based on release‐ or contact‐killing. The studies reported a complete eradication of all bacteria in less than 5 min (3‐log reduction) [60], after 6 h (6‐log reduction) [61], 100% inhibition after 24 h (8‐log reduction) [62] (5‐log reduction) [63], 100% inhibition after 24 h (7‐log reduction) [64], 7‐log bacterial reduction [65], 6‐log bacterial reduction [66], 3‐log bacterial reduction [67], almost 100% (3‐log reduction) [68] and between 6‐log reduction [69] and 1‐log reduction [70–76] antibacterial efficacy. A summary of the essential data presented about polyurethanes containing QACs is provided in Table 4.

In this review, it was also investigated whether the antimicrobial properties of QACs are connected to their position in the structure. In a majority of the reviewed studies, QACs are positioned in a pendant group. There is only one study with QACs positioned in the terminal groups (that study reported a bacterial reduction of 100% [4‐log reduction] [49]) and four compounds with the QACs positioned in the backbone (reporting a bacterial reduction of 29% [55] and 25% [56] with no ZOI, and a bacterial reduction of > 3 log [57] with a ZOI, and 3‐log reduction [68, 71, 73], and 1.7‐log reduction [75] when the ZOI was not investigated). There is one study where the QACs were positioned in both backbone and terminal group (89.7% [58] bacterial reduction with a ZOI) and there is one study where the QACs were positioned in both the backbone and pendant group (bacterial reduction of 100% [7‐log reduction] [64]). A summary of the different log reduction related to the location of the QAC is provided in Table 3.

Due to the limited number of studies on compounds in which the QACs are not part of the pendant group, establishing a link between the structure and antimicrobial properties of QAC‐containing polyurethanes is challenging. Based on the analysis of the published data, it appears that such a link does not exist.

##### 3.2.1.3. Guanidine

Guanidine moieties in polyurethanes are known to show robust, broad‐spectrum antimicrobial activity [175]. Published research demonstrated that the positive charges of the guanidinium cation interact with the negatively charged bacterial cell wall through electrostatic attraction, resulting in the leakage of the bacteria′s intracellular contents and ultimately leading to bacterial death [147, 176]. In contrast, electrically neutral mammalian cell membranes are not damaged by the guanidinium cation [177, 178].

Guanidine‐containing polyurethanes have been shown to kill up to 3 log of bacteria by Peng et al. [77] or more than 2 log by Zhang et al. [78]. Zhang also observed that guanidine incorporated in anionic films showed no antibacterial activity in contrast to cationic films. Several studies on guanidine‐containing polyurethanes attributed the antimicrobial properties not to a contact‐killing mechanism, but to the antiadhesive abilities of guanidine. Richards et al. [79] propose that the positive charge of the guanidine can inhibit microbial adherence by repelling the bacteria. No bacteria were found after 60 min (6‐log reduction) on the guanidine/PU–catheter they designed. According to Gharibi and Agarwal [80], their films could prevent bacterial adhesion thanks to the hydrophilicity of the polar amine and guanidinium groups, which build hydrogen bonds with the water and form a hydration layer that can prevent bacterial attachment. They reported a killing efficiency of 3 log. A summary of these results has been provided in Table 5.

##### 3.2.1.4. Other Synthetic Organic Compounds

###### 3.2.1.4.1. Other Cationic Compounds

There are other cationic, bactericidal compounds like the polycation polyethyleneimine (PEI) [81, 149] which can disrupt the bacterial cell membrane, resulting in cell lysis and cell death [81–83]. Additionally, PEI shows bactericidal activity via electrostatic forces [81, 179] and via the positively charged amino groups [82]. This review found that PEI is primarily used to modify polyurethane as polymer brushes. The brushes, due to their chemical structure and dynamic motion, can prevent adhesion, bacterial proliferation, and biofilm formation [81–83]. Gultekinoglu et al. reported that PEI brushes prohibit bacterial adhesion by 2 log in 2015 [81] and reduced the bacterial amount by 2 log after 24 h in 2017 [82]. Gultekinoglu et al. [83] also used a single‐cell force spectroscopy to investigate the rupture forces between *Eschirichia coli* K‐12 and alkylated PEI grafted polyurethane surfaces, showing that alkylated high molecular weight–PEI brushes show the lowest rupture force against single bacterial cells.

Boron compounds are known for both bactericidal and bacteriostatic mechanisms, and there is no consensus in the literature about the mode of action. One possible explanation is that the cationic moiety around the positively charged Boron atom can interact with the negatively charged bacterial membrane, which breaks it and kills the bacteria [84, 180]. Sürdem et al. [84] made boric acid–incorporated polyurethane foams, which showed a bacterial reduction of up to 4.8‐log reduction.

###### 3.2.1.4.2. Other Nonionic Compounds

Some nonionic organic compounds also possess antimicrobial properties. Glutaraldehyde‐impregnated polyurethane was capable of reducing the bacterial amount by over 4 log [85], polyurethanes containing surfactant‐modified random nanosilicate platelets reduced bacteria by up to 100% (5 log) [86] and polyurethanes with the broad‐spectrum antibiotic pendant benzisothiazolinone (BIT) exhibited a bactericidal reduction of up to 1.1 log and no zone of inhibition [87]. The primary outcomes of the other cationic and nonionic compounds have been summarized in Table 6.

#### 3.2.2. Natural Compounds

Several compounds in nature have been reported to have antimicrobial properties, and for many years, natural antimicrobial compounds were among the most approved by the FDA [180].

##### 3.2.2.1. Chitosan

A well‐known natural antimicrobial compound is chitosan, a cationic polysaccharide that can interact with the negatively charged bacterial cell membrane. By destroying it, the bacterial components leak out [88, 89, 96, 146]. Polyurethanes containing chitosan could kill all bacteria after 18 h (> 5‐log reduction) [88] or between 99.99998% (6.7‐log reduction) [89] and 20% [90–94, 181] of all bacteria. Two studies by Kara et al. [95] and Arevalo et al. [96] claimed that, in addition to the contact‐killing mechanism, chitosan also improves the hydrophilicity of the polymer through its polar groups and thereby hinders bacterial adhesion. They reported a 5 log [95] and 54% [96] bacterial reduction. The primary outcomes of the manuscripts discussing chitosan‐containing polyurethanes are summarized in Table 7.

##### 3.2.2.2. Chitin, Peptides, and Curcumin

Chitin is a natural amino polysaccharide similar to chitosan [123]. Chitin can trigger bacterial flocculation, which presumably kills the bacteria by cutting off nutrients and oxygen [97]. In contrast to chitosan, chitin lacks the number of polycationic amines that interact with the negatively charged cell surface, so it is less capable of inhibiting bacterial growth [97]. Zia et al. [97] investigated hydroxy‐terminated polybutadiene–chitin–based polyurethanes, which, depending on the chitin concentration, inhibited bacterial growth underneath the sample in a zone of inhibition test or stopped bacterial growth in an 8‐cm zone around the sample. Peptide‐modified polyurethane surfaces seem to disrupt the bacterial membrane, similar to charged amines or QACs, according to microscopy studies by Lu et al. [98] and Peng et al. [99]. They both reported no leaching behavior according to inhibition zone tests, as well as up to 3‐log bacterial reduction [98] and antibiofilm abilities that remained for at least 5 days [99]. Curcumin is a hydrophobic polyphenol that is capable of damaging the cell membrane, among other antimicrobial mechanisms [100]. Turbidity measurements done by Abdelbasset et al. [100] showed a bacterial reduction of up to 1.3 log in 24 h for curcumin incorporated in polyurethane.

##### 3.2.2.3. Other Natural Compounds

Other natural compounds that were combined with polyurethane and tested consist of a combination of two different essential oils (alpha‐terpineol and cinnamaldehyde), resulting in a 6‐log bacterial reduction [101], lysostaphin with a 4‐log reduction [102], the biobased chain extender, 2,5‐diformylfuran dioxime, with 100% reduction (4‐log reduction) [103], *Antheraea mylitta* silk fibroin with 70% microbial adherence reduction and a zone of inhibition of up to 1.85 cm [104], acylase with a 60% bacterial reduction [105] and terpenes with a bacterial reduction of up to 60% for menthol [106].

The primary outcomes of the manuscripts discussing polyurethanes containing natural compounds are outlined in Table 8.

### 3.3. Antiadhesive Modifications

In contrast to compounds that kill bacteria upon contact, antiadhesive modifications are utilized to prevent bacteria from attaching to a surface. This can have a high impact on preventing an infection as bacterial adhesion is the necessary first step in biofilm formation [182].

#### 3.3.1. Surface Modifications

A common way to prevent bacteria from adhering is to alter the topography of a surface, such as adding micro‐ or nanostructures inspired by nature [111, 183]. Structured surfaces reduce the area where bacteria can adhere, especially when the surface features are smaller than the size of the bacteria themselves [107, 160, 161]. The structure can also change the surface energy and surface wettability [161]. Certain nonwetting topographies, such as the Sharklet micropattern, are superhydrophobic, creating air pockets on the surface that impede bacterial interaction and thereby prevent biofilm formation [107, 109, 184]. Structured surfaces can also kill bacteria by mechanically rupturing the bacterial membranes [108, 184, 185]. Siddiquie et al. [107] used a femtosecond laser processing to provide a polyurethane with surface micro‐/nanotexturing. A study using a spinning disk confocal microscope reported that texturing could reduce bacterial adhesion by up to 1.4 log after 16 h. Ganbaatar et al. [108] investigated nanoline–array surfaces with different spacing, reporting up to 79% bacterial reduction. May et al. [109] tested a Sharklet micropattern made by thermal embossing, which reduced bacterial colonization by up to 71%. Tan et al. [110] investigated thermoplastic polyurethanes with a micro‐nanostructure surface that could reduce bacteria attachment by 66.7%. Gao et al. [111] investigated ordered hemisphere patterns, and the most notable result was achieved with the 2‐*μ*m patterned PU film, 37.5% bacterial reduction. Although adding a topography to a surface can reduce bacterial adhesion, smoothing a surface can promote bacterial attachment. Mrad et al. [112] used a new plasma decontamination treatment to graft oxygen and nitrogen species onto the surface, but the resulting smoothing of the surface led to a slight but significant bacterial increase for the treated PU of up to 0.57 log. Restivo et al. [113] investigated if modifying the thermoplastic polyurethane topography by brush and bar coater deposition techniques would affect the adherence of bacteria. *E. coli* adhered independently of the deposition method, and the viability of *Staphylococcus aureus* increased by 20% after brush‐coating and reduced by 25% after bar‐coating. The primary outcomes of the surface‐modified polyurethanes are summarized in Table 9.

#### 3.3.2. PEG

PEG is a polymer often used due to its antiadhesive properties, its nontoxicity, and hydrophilicity [186–188]. PEG lowers the surface energy of a material and can be a steric hindrance when used in the form of brushes [133, 189]. Due to its hydrophilicity, PEG can also form a hydrated layer on the surface [116, 132, 189]. Cao et al. [114] reported a 2.3‐log bacterial reduction for both polyurethane containing PEG and polyurethane containing poly[(ethylene oxide)‐co‐(ethylene carbonate)] (PEOC). Meng et al. [115] modified a polyurethane membrane with PEG monomethyl ether and reported a bacterial reduction of up to 1.5 log. Cao et al. [142] also investigated PEG‐modified polyurethane and reported a reduction in bacterial adhesion of over 1.4 log. Li et al. [116] designed a polyurethane film containing PEG, reporting a 17% bacterial reduction.

#### 3.3.3. Fluor

Fluorinating a polymer lowers its surface tension [117], improves hydrophobic behavior and biostability [36, 127, 190, 191], and inhibits biofilm formation [160] by several mechanisms, including preventing bacterial attachment [192] and disrupting bacterial energy production and metabolism [193]. Qiao et al. [117] synthesized a series of fluorinated polyurethanes and showed a gradual decrease of bacterial adhesion with an increasing amount of fluor. They reported a bacterial reduction of over 1 log.

#### 3.3.4. Other Antiadhesive Compounds

Inspired by the natural compound borneol, various derivatives were combined with polyurethane to impart antimicrobial properties [118, 119]. Chen et al. [118] used isobornyl acrylate to make a coating that showed excellent antiadhesion performance. Their antiadhesive testing showed no attached bacteria (4‐log reduction) at a borneol acrylate concentration of 54%. Wu et al. [119] also made a coating with a borneol derivative (isobornyl acrylate), which inhibited bacteria by up to 89.3% according to plate counting. Another antiadhesive compound that was used with polyurethane was N‐vanillylnonanamide (a synthetic analog to capsaicin), but Villa et al. [120] reported that adding this compound to polyurethane did not change the bacterial adhesion in any significant way.

Table 10 provides a summary of the principal outcomes for polyurethanes modified with antiadhesive compounds.

**Figure 4:**
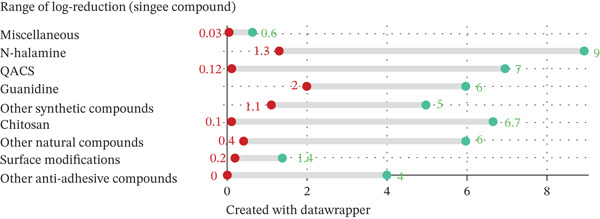
Schematic representation summarizing the ranges of reported in vitro log‐reduction values in studies of polyurethane materials functionalized exclusively with a single antimicrobial compound, categorized by antimicrobial compound category.

An overview of the log‐reduction values in the studies of polyurethane materials exclusively functionalized with a single antimicrobial compound is provided in Figure 4.

### 3.4. Polyurethanes Modified With Several Compounds

Many studies found in the literature search for this review combined two compounds in a polyurethane. The incorporation of two compounds with contact‐killing mechanisms can enhance the antimicrobial properties of a polymer; however, combining antiadhesive and contact‐killing compounds may offer an even more effective synergistic approach. Dead bacteria may accumulate on the surface of contact‐killing materials, potentially preventing the interaction of viable bacteria with the antimicrobial matrix. The incorporation of antiadhesive mechanisms can mitigate this issue, thereby preserving the efficacy of the contact‐killing function [194].

#### 3.4.1. Synthetic Compounds

##### 3.4.1.1. Halogen‐Containing Compounds

Lin et al. [36] tested fluorinated and unfluorinated N‐halamines to investigate if the antiadhesion ability of fluor could enhance the antibacterial properties of the N‐halamines. Unexpectedly, the antimicrobial efficiency of the unfluorinated N‐halamine was reported to be much higher (5‐log reduction in contrast to 3‐log reduction after 15 min). Lin et al. explained the lower efficiency of the fluorinated form with its lower solubility in the polymer, resulting in the compound forming domains on the polyurethane surfaces, so they had less contact with the bacteria. In contrast, Li et al. [121] investigated chloramine precursors containing QA units, which boosted their bactericidal activity with a bacterial reduction of up to 72.1%. Li et al. also proved the regenerability of the surfaces.

Rogalsky et al. [122] added polyhexamethylene guanidine hydrochloride (PHMG‐Cl) to a commercial polyurethane. Polyurethanes containing more than 3% PHMG‐Cl showed biocide release, whereas nothing eluted at lower concentrations. A total inhibition of all bacteria was reached with a PHMG‐Cl content of 3% (4‐log reduction).

Table 11 provides a summary of the principal outcomes from studies on polyurethanes incorporating halogen‐containing compounds together with a secondary component.

##### 3.4.1.2. Quaternary Compounds

Xie et al. [123] investigated quaternized chitin and chitosan tethered to polyurethane, which are not only contact‐killing but can also form a hydration layer due to their amphiphilic structure, thereby preventing bacterial adhesion. The quaternized chitin matrix demonstrated a killing efficacy of up to 66%, whereas the quaternized chitosan matrix showed a killing efficiency of up to 1.7 log. Chen et al. [124] immobilized quaternary ammonium chitooligosaccharide over a fibrous PU membrane and reported an antibacterial efficacy of up to 75.3% after 24 h. Huang et al. [125] grafted polymerized imidazolium salt chitosan and polymerized 2‐methacryloyloxyethyl phosphorylcholine on polydopamine/PEI–modified polyurethane. The synergistic mechanism of the QACs in the imidazole rings, the chitosan, and the antiadhesion ability of the zwitterionic PMPC resulted in an antibacterial rate of 3.2 log.

Several studies investigated combining PEG and QACs in a polyurethane matrix. In 2007, Kurt et al. [126] integrated cationic contact–killing alkylammonium (QAC) and either trifluoroethoxy or PEGylated side chains in polyurethane coatings, and all the coatings had an indistinguishable 100% bacterial (3.6‐ to 4.4‐log reduction) effectiveness after 30 min without a zone of inhibition. In 2008, Pinar Kurt and Kenneth [127] synthesized a coating containing QACs and fluor. They reported a 3.6‐ to 4.4‐log bacterial reduction in 30 min. Wang et al. made a polyurethane coating with quaternary ammonium and PEG‐like side chains and reported > 4‐log bacterial reduction [128]. Polyurethane films containing a QAC upper layer and an antiadhesive PEG sublayer show promising antimicrobial properties: Zhang et al. [129] reported that more than 4‐log bacteria were killed without inhibition zones, and He et al. [130] reported a reduction of 4 log. In contrast, a polyurethane containing QACs and PEG made by Aydin [131] et al. showed a bacterial reduction of up to 1.3 log. Simply tethering quaternary ammonium to PEG‐containing urethanes, as Coneski et al. [132] did, resulted in a biocidal activity of 1 log and a reduction of microbial adherence of 60%. Du et al. [133] used a combination of methoxypolyethyleneglycols (MPEG), which can form a network that prevents bacterial adhesion, and QACs formed with guanidine to create a double positive charge. The coating had an antibacterial efficiency of up to 98.2% (1.7‐log reduction). Zheng et al. [134] grafted QACs, PEG, and MPEGs on a plant oil–based waterborne polyurethane material and achieved a bacterial reduction of 1.1 log. Guo et al. [135] investigated polyurethanes incorporating quaternary ammonium salt and PEG with an 82% bacterial reduction. Gupta et al. [136] synthesized polyurethanes including QACs and the antiadhesive PPG and PEG and reported zones of inhibition of up to 8 mm.

QACS were also combined with several other antiadhesive compounds. Sharma et al. [137] covalently modified polyurethane urological implants with polydimethyl siloxane, lowering surface energy and generating a hydrophobic barrier. They incorporated quaternized moieties in branched PEI and reported a bacteria reduction of up to 3 log. Liu et al. [138] investigated amphiphilic carbonaceous particles with quaternized polyethyleneimine as pendant groups, blended with thermoplastic polyurethane by a carrier dispersion strategy, and reported a 1.5‐log loss of bacteria viability. The polymer demonstrates antiadhesion characteristics attributed by the authors to the antimicrobial action of the exposed ACPs‐QPEI, which effectively eliminates bacterial cells upon their attachment to the surface. Jiang et al. [139] prepared quaternized ammonium–modified methyldiethanolamines (QMDEA)–containing polyurethanes containing the antiadhesive polymer polyvinylpyrrolidone. At a concentration of 3% of the QMDEA, the coating had an antibacterial efficiency of 3 log, and no zone of inhibition was observed. Sulfobetaines are zwitterionic compounds usually containing a QAC as their positive charge. They can form a hydration layer and prevent bacterial attachment [195]. Also, Shi et al. [140] investigated polyurethane with bisfuran‐based polyamide polymers with various functional groups, including allyl quaternary ammonium cations, decyl quaternary ammonium cations, and sulfonate betaine, and reported > 2 log even after seven cycles of use. Zhu et al. [141] grafted zwitterionic sulfobetaine methacrylate on polyurethane catheters. The coatings could eliminate over 2 log of bacteria after 7 days. Cao et al. [142] also investigated sulfobetaine‐modified polyurethane and reported a reduction in bacterial adhesion of over 1.4‐log.

Levana et al. [143] embedded thermoplastic polyurethane with quaternary ammonium salts contained in montmorillonite (MMT) clay, a nanoclay that inhibits bacterial adhesion. The film demonstrated up to 85.34% killing efficiency. Jia et al. [144] made a dispersion of cationic waterborne polyurethane containing quaternary ammonium salts and siloxane. They inferred that due to siloxane, the polymer chains could disrupt the bacterial membrane even more effectively. The dispersion showed inhibition zones up to 13.71 mm.

To provide an overview of the principle outcomes of the manuscripts investigating polyurethanes containing QACs in combination with another compound, a summary is presented in Table 12.

##### 3.4.1.3. Guanidine

Liu et al. [146] modified a polyurethane coating with chitosan biguanide hydrochloride and reported an antibacterial rate of up to 1 log. Gharibi and Agarwal [147] functionalized polyurethane with a contact‐active polyguanidine bactericidal agent and oxidized dextran as an antiadhesive agent. The polyurethane exhibited a 100% killing efficiency (8‐log reduction). Hyaluronic acid is one of the most hydrophilic natural compounds [196] and is capable of repelling bacteria with its negative charge by the electrostatic repulsive force [197]. Yuan et al. [148] reported that polyhexamethylene guanidine/hyaluronic acid multilayer films showed inhibition rates of up to 4 log. These primary outcomes are summarized in Table 13.

##### 3.4.1.4. Other Synthetic Compounds

Sharma et al. [149] investigated polydimethylsiloxane modified thermoplastic polyurethane incorporating branched polyethyleneimine and poly(2‐ethyl‐2‐oxazoline). Although PEI is contact‐killing, poly(2‐ethyl‐2‐oxazoline) is a bioinspired polymer with antiadhesive properties. They report a 4‐fold log reduction in the planktonic growth. Liu et al. [150] combined the positively charged contact‐killing antimicrobial agents dimethyloctadecyl[3‐(trimethoxysilyl) propyl] ammonium chloride, benzalkonium chloride, and octenidine dihydrochloride in a polyurethane coating with more than 2‐log bacterial reduction. Tetracycline is an antibiotic that blocks the peptide synthesis of tRNA in ribosomes [151, 198]. Chung et al. [151] integrated tetracycline as a contact‐killing compound, as well as dimethylolpropionic acid and polyol to enhance the hydrophilicity of the PU and thereby the antibacterial activity. They reported a bacterial reduction of up to > 8.3 log. Gharibi et al. [152] made polyurethanes with methoxysilane functional aniline tetramers, which can interact with the anionic cell membranes. They reported that the polyurethane/siloxane membranes had higher antimicrobial activity than the membranes without siloxane, reducing nearly 100% (inoculum size was not defined in this study, so how big the log reduction was unclear) of all bacteria. Another biocidal compound is triazole and its derivatives [153]. It has been shown that the compounds can inhibit DNA gyrase, glucosamine‐6‐phosphate synthase, dihydrofolate reductase, and SecA ATPase, all of which are essential bacterial proteins [199]. Wen et al. [153] coated polyurethanes with the antiadhesive polymer PVP via triazole links. The membranes showed up to 67% reduction in bacterial attachment. Table 14 summarizes the primary outcomes of the manuscripts discussed in the paragraph above.

##### 3.4.2. Natural Compounds

The natural polymer heparin can bind calcium, which suggests that it acts as a chelation agent and, thereby, removes necessary cations from bacteria [154, 155, 200]. Kara et al. integrated chitosan and heparin into polyurethane in two studies. In 2015, they reported a > 3.5‐log reduction [155] and in 2016, they reported that all bacteria were killed on the sample after 24‐h inhibition (6‐ to 7‐log reduction) [154]. Similarly, Li et al. [156] integrated chitosan azide and heparin azide into polyurethane films with a bacterial reduction of 1.1 log. Luo et al. [157] used the self‐polymerization of dopamine to attach chitooligosaccharide to the surface of a polyurethane membrane and reported an antibacterial efficacy of 48.78%. Hu et al. [158] integrated the photosensitizer phthaloyl phthalic acid and the antimicrobial agent caffeic acid for a synergistic effect under sunlight and reported a 2.1‐log reduction. A summary of the primary outcomes of the polyurethanes containing natural compounds combined with another compound is summarized in Table 15.

##### 3.4.3. Antiadhesive

Surface modifications are independent of other antimicrobial modifications and can therefore be easily combined [183]. Kim et al. [159] designed films with variously sized holes (ranging from 0.3 to 4 mm) and coated with the antiadhesive, zwitterionic polymer 2‐methacryloyloxyethyl phosphorylcholine. After 18 h, the surface could inhibit *S. aureus* colonization by 82%. Xu et al. [160] used a combination of submicron pillars and fluorocarbon to reduce bacterial adhesion by up to 75% according to SEM measurements. The films that showed biofilm formation had a high content of fluorocarbon, which most likely lowered the mechanical strength, leading to defects in the pillars. In 2017, Xu et al. [161] also made polyurethane‐bearing ordered pillar topographies as the top layer with an S‐nitroso‐N‐acetylpenicillamine (SNAP, NO donor) doped sublayer underneath. They report an 88% bacterial reduction rate according to turbidity measurements. Walter et al. [162] combined PPG and PEG in a polyurethane membrane. They claim that the glycols enhance the hydrophobicity of the membrane, providing intimate contact with the biocidal urethane moieties. They test this using an inhibition zone test, which shows zones up to 16 mm. Ajit Walter et al. [163] also synthesized a series of polyhedral oligomeric silsesquioxane‐based poly(urea‐urethane–imide) membranes. The imide moiety enhanced surface hydrophilicity thereby increasing the number of bacteria that come into contact with the surface, where the urea moieties can kill the bacteria. The antiadhesive silesquioxane was used to reduce surface energy. According to SEM images, the membranes reduced bacterial attachment by up to ~2‐log. Disk Diffusion assays showed zones reaching up to 15 mm. Rossie de Aguilar [164] made urethanic materials containing polydimethylsiloxane and polyoxometalate (phosphotungstic acid), which suppressed bacteria attachment for 4 h of contact. Table 16 offers an overview of the principle outcomes discussed in the preceding section.

### 3.5. In Vivo Studies of Organic Polyurethanes

Out of the 135 studies identified in this review′s literature search, only 13 conducted in vivo testing (Table 17), and just six focused on antimicrobial in vivo testing. Four of these studies investigated quaternized materials. The highest reduction was reported by Peng et al. [70] for mixed‐charge zwitterionic polyurethane coatings with cationic quaternary amine groups in their backbone. The coatings showed a bactericidal ratio of up to 3 log in vitro and a 2–3 log reduction in a reoperative infection model in the subcutaneous tissue of SD rats. Xie et al. [123] investigated quaternized chitin and chitosan tethered to polyurethane with a killing efficacy of up to 66% for chitin and up to 1.7 log for chitosan in vitro and up to a 3‐log reduction after subcutaneous implantation in mice. Liu et al. [138] constructed amphiphilic carbonaceous particles with quaternized polyethyleneimine as pendant groups, blended with thermoplastic polyurethane by a carrier dispersion strategy, and reported 1.5‐log bacterial reduction in vitro. After implanting the polyurethane subcutaneously into mice, it retained over 1‐log antimicrobial efficacy after 2 months. Jiang et al. [139] made QMDEA as pendant groups of polyurethanes blended with antiadhesive polymer polyvinylpyrrolidone. In vitro testing showed an antibacterial efficiency of 3 log, and no zone of inhibition, and subcutaneously implanted into infected mice showed a bactericidal ratio of 87.48%. Dhyani et al. [101] made a medical‐grade polyurethane coating blended with two different EO components (alpha‐terpineol and cinnamaldehyde) and reported a 6‐log bacterial reduction in vitro. In an in vivo full‐thickness porcine burn model with a total of six burns, bacteria were reduced by over 5 log after 2 and 3 days. Finally, Lu et al. [98] investigated the antibacterial efficiency of peptide polymer–modified thermoplastic PU surfaces with the peptide in the pendant groups. They reported bacterial reduction of up to 3 log in vitro and 1.27 log with a subcutaneous implantation infectious model.

The observed discrepancies between in vitro and in vivo antimicrobial efficacy results can be attributed to the translational gap in clinical implementation. This gap arises from the lack of standardized protocols for in vitro and in vivo models, the inherent complexity of in vivo models, and a lack of validation between in vivo tests and clinical results [201, 202].

## 4. Discussion

This systematic literature review describes a comprehensive overview of organic, antimicrobial surface–active polyurethanes, the compounds used to provide them with antimicrobial properties, their antimicrobial effectiveness, and the mode of action. A variety of materials, both synthetic and natural, can be incorporated into polyurethanes to provide them with contact‐killing or bacteria‐repelling properties with varying efficacy. The analysis highlighted QACs, N‐halamines, and chitosan as the most promising antimicrobial agents, with their efficacy most extensively supported by the existing literature [33, 36, 60, 62, 88, 89]. To the best of our knowledge, none of these compounds have yet been implemented in clinical settings. The potential reasons for this lack of clinical translation are discussed in detail below.

The studies included in this review consistently report that unmodified polyurethanes exhibit no inherent antimicrobial activity, with the exception of two studies by Walter et al. [162, 163] which suggests that urethane moieties themselves possess biocidal properties. However, in both cases, the polyurethanes were concurrently modified with additional compounds known to influence antimicrobial performance, namely, polypropylene glycol and PEG in one study [162], and silsesquioxane in the other [163]. Notably, neither study included control experiments isolating the antimicrobial effect of the urethane moieties alone, nor did they provide a mechanistic rationale or literature citations supporting the claimed intrinsic biocidal activity. Consequently, based on the available evidence, it must be concluded that polyurethane does not exhibit antimicrobial properties in the absence of incorporated antimicrobial agents. The bacterial reduction of these polyurethane materials was also notably low (Figure 4).

Among the various material technologies investigated, the majority of the published polyurethanes exhibited a range of antimicrobial efficacy, from limited to highly effective. QACs emerged as both the most extensively studied (as shown in Figure 2) and among the most promising contact‐killing antimicrobial agents incorporated into polyurethane matrices. Several of these matrices demonstrated 100% antimicrobial reduction, most notably reducing 3 log in less than 5 min [60] and 8 log in 24 h [62]. However, on the lower end, they only showed up to 25% reduction (0.13 log) [56] (Figure 4). QACs have been incorporated into various segments of the polyurethane structure (its backbone, pendant groups, and terminal ends) and have been utilized in both eluting and non eluting antimicrobial systems. However, due to the significant variability in chemical structures and in vitro testing methodologies across these modified polyurethane matrices, a direct comparison to determine the most effective QAC‐functionalization strategy remains challenging. Although the wide range of research brings many possibilities, the widespread use has also led to an increasing bacterial resistance against these compounds, making additional research progressively important [54]. Prior to clinical implementation, antimicrobial compounds must undergo rigorous regulatory evaluation to ensure safety and efficacy. A critical requirement is the demonstration of noncytotoxicity, which was only tested in some of the QAC studies in this review. Evaluation of antimicrobial activity in relevant animal models represents a key subsequent step in the translational process. Among all antimicrobial in vivo studies included in this review, the majority investigated quaternized compounds with a reduction of up to 3 log [70]. This positions QACs as the most advanced candidates among the evaluated agents in terms of potential clinical translation. However, to the best of the authors′ knowledge, no QAC‐based materials have yet received approval for use in medical devices. This may be attributed to the limited number of in vivo investigations identified in this review (*n* = 4), the broader translational challenges associated with bridging preclinical in vivo findings to clinical implementation, as well as many other regulatory hurdles. In the EU, as an example, the medical device regulation requires in‐depth preclinical data and risk analysis, as well as focused claim language and experimental data to verify it.

N‐halamines represent the second most extensively studied class of antimicrobial agents (Figure 2) in this review. These compounds show great potential, being capable of killing all bacteria on a matrix (up to 8–9 log [36] and 6–7 log [33] in 5 min). In contrast to other antimicrobial agents, N‐halamines offer the distinct advantage of a regenerable biocidal mechanism, which can be restored through exposure to free halogen sources such as bleach [41]. However, within the scope of the medical applications addressed in this review, such regeneration is generally limited to temporary devices, such as catheters, where periodic halogen treatment is feasible. Despite the highly promising antimicrobial performance of N‐halamine‐functionalized polyurethanes (Figure 4) reported in this review, the absence of in vivo studies remains a significant barrier to their clinical translation.

Several other cationic compounds also demonstrated promising antimicrobial activity, like guanidines, which achieved a bacterial reduction of up to 6 log [79] and PEI, demonstrating up to a 2‐log bacterial reduction [81, 82] when incorporated into polyurethane. However, the limited number of studies (Figure 2) investigating these compounds without another antimicrobial compound incorporated into the polyurethane—only four studies for guanidines and three for PEIs in vitro and none in vivo*—impedes* any definitive conclusions regarding their efficacy or potential. The current evidence, although encouraging, underscores the need for further systematic research to fully assess their performance and applicability. Other antimicrobial compounds like surfactant‐modified random nanosilicate platelets (5 log) [86], boric acid (4.8 log) [84], glutaraldehyde (> 4‐log reduction) [85], and BIT (1.1 log) [87] showed very promising antimicrobial properties in only one study, necessitating further research to both confirm the antimicrobial properties in vitro as well as prove those properties translate into in vivo.

Nonsynthetic compounds represent another major class of antimicrobial agents, of which chitosan is the most extensively studied (Figure 2), with reported bacterial reductions of up to a 6.7 log [89] and 5 log [88]. The absence of in vivo studies of chitosan‐incorporated polyurethane remains a significant barrier to their clinical translation. Among the other natural compounds, some of the most remarkable results were reported for alpha‐terpineol and cinnamaldehyde (6 log) [101], lysostaphin (4 log) [102], 2,5‐diformylfuran dioxime (4 log) [103], peptides (3 log in vitro and 1.3 log in vivo) [98], curcumin (1.3 log) [100] showing very promising antimicrobial properties and necessitating further research.

Antiadhesion strategies for polyurethane‐based materials encompass a range of strategies. Among these strategies, surface modifications that impede bacterial adhesion have been investigated the most extensively (Figure 2). Although a variety of surface engineering techniques have been explored, none have demonstrated consistently high levels of bacterial reduction when applied in isolation. Except for one study reaching a 1.4‐log reduction [107] in bacterial adhesion, all others reported < 1‐log reduction (Figure 4). A compound that demonstrates much higher antimicrobial activity is PEG with up to a 2.3 log [114] and 1.4 log [116] reported bacterial reduction. Although promising, with only four studies in this review, further research will be needed. Apart from PEG, the only antiadhesion compound that demonstrated potential was borneol, as evidenced by a single study reporting a 4‐log bacterial reduction [118]. Given the robust methodology employed to assess antimicrobial properties in that study, these findings are promising. Additional research is required to determine whether borneol exerts true antiadhesive effects or if the observed reduction is due to contact‐killing activity, as the latter was not experimentally evaluated but merely inferred [118].

Antiadhesion strategies for polyurethane can reduce the bacterial amount on a surface, but antiadhesive compounds seem to show the most promise if combined with one or more contact‐killing compounds. Antiadhesive strategies have been investigated far more in combination with other compounds than alone (Figure 2). This combination prevents dead bacteria from accumulating on the surface of contact‐killing surfaces, so they can retain their contact‐killing properties for a longer time [194]. The highest antimicrobial reduction was observed for tetracycline, dimethylolpropionic acid, and polyol incorporated in polyurethane with > 8.3‐log [151] reduction, for a combination of polyguanidine and oxidized dextran with 100% bacterial reduction (8 log) [147] and for chitosan and heparin grafted on polyurethane (6 to 7‐log reduction) [155]. Although combining two compounds in a matrix can enhance their antimicrobial activities, some studies have also shown that the two compounds can interfere with each other′s mode of action. Lin et al. [36] observed that adding fluor to their N‐halamine actually reduced its antimicrobial activity. They explained the lower efficiency of the fluorinated form with its lower solubility in the polymer, making the compound form domains on the polyurethane surfaces, so they had less contact with the bacteria. Most studies that combined two antimicrobial compounds in polyurethane did not explore whether their antimicrobial effectiveness would be enhanced or reduced compared with the same polyurethane containing just one of those compounds. As a result, it′s not possible based on current literature to determine whether adding two antimicrobial compounds to polyurethane results in more effective antimicrobial properties.

Even within the same class of antimicrobial agents, meaningful comparisons are complicated by differences in the mode of incorporation into the polyurethane matrix. Covalent attachment to distinct regions—such as the backbone, pendant side chains, or terminal groups—can already significantly influence antimicrobial performance, but physical blending of antimicrobial agents into the polymer introduces additional challenges. A key challenge is that the blended compound has to be uniformly dispersed and stable [203]. Low molecular weight compounds may uncontrollably leach out of the matrix over time, which needs to be avoided [204], but at the same time, effective antimicrobial action requires proximity to the polymer surface [205]. All these reasons make the comparison of covalently bonded and physically blended antimicrobial compounds difficult.

There are numerous approaches involving both synthesis and blending for the design of contact‐killing antimicrobial polyurethanes. The presentation of antimicrobial groups on the surface is critically important, as are the inactive portions of the polyurethane. This can be enhanced by introducing antiadhesive functionality. However, all these factors must be balanced with considerations for synthetic and fabrication ease. Translating this into a scalable synthetic process is challenging [206, 207]. Additionally, surface structuring of the polyurethane materials can further enhance antimicrobial performance in the short‐ and long‐term.

Although great progress has been made in this research topic, many challenges remain. During the data extraction of this review, it became apparent that comparing the antimicrobial results across different studies is challenging, not only because of differences in chemical composition and material technology but also due to the lack of standardized parameters. These parameters include broth type [172], inoculum size [45], and strain of the used bacteria [91], among others. To give some exact numbers: 19 studies in this review omitted the broth type they used [35, 37, 38, 43, 56, 70, 74, 75, 77, 80, 89, 92, 97, 98, 126, 127, 131, 133, 138] and 11 studies did not mention their inoculum size [58, 70, 74, 80, 97, 99, 100, 123, 124, 127, 157]. Additionally, most studies included in this review reported their antimicrobial results as percentage reduction and not on a logarithmic scale, so the results needed to be recalculated for this review. It is much easier to understand and compare antibacterial effectiveness on a log scale, but this approach appears to be underutilized in the current literature.

Terminology is also not used consistently in the studies of this review. Terms like bacteriostatic, bactericidal, as well as antiadhesion or contact killing are used to describe different observations. For example, Liu et al. [138] claimed their QACs incorporated in polyurethane have an anti‐adhesive mechanism because they kill the bacteria that touch the compounds, whereas most studies in this review consider such compounds contact killing. The absence of standardized terminology in scientific literature hinders the effective comparison of results, complicates discussion surrounding the optimization of promising compounds, and contributes to the widening of the translational gap between laboratory research and clinical application.

The terms bacteriostatic and bactericidal are misused even more often than contact‐killing and anti‐adhesion because they are more based on convention than on clinical principles [17]. The strict definition of “bactericidal” compounds kill bacteria, and “bacteriostatic” compounds prevent their growth (the stationary phase of growth) strictly applies only to laboratory environments [16]. According to the official definition, bactericidal antibiotics have a ratio of MBC‐to‐MIC of ≤ 4 and bacteriostatic agents of > 4 [16], but these definitions are somewhat arbitrary and often not linked to the outcome in vivo [17]. In reality, those categories are not that strict, and bacteriostatic compounds kill some bacteria, whereas bactericidal compounds do not kill all bacteria [16]. Many studies in this review that claim a bactericidal/bacteriostatic mechanism do not utilize tests (e.g., MBC or MIC) to conclusively investigate and support these results.

Other antimicrobial tests utilized in this review also do not seem suited for the purpose stated in the studies either. For example, Walter et al. [162] claimed to use PPG and PEG to provide intimate contact with the biocidal urethane moieties. They tested this with a zone of inhibition test, which assesses the release of antimicrobial compounds. Other polyurethanes are supposed to be both eluting, antiadhesive, and contact‐killing, but are only tested by the zone of inhibition test [97]. Turbidity measurements were also often utilized without additional testing, even though they detect growth only at the late exponential phase, making turbidity measurements limitedly efficient for studies focusing on low cell numbers [208]. Turbidity measurements can also not differentiate between living and dead cells.

The compounds discussed in this review often have different alleged modes of action. There is frequently a lack of consensus in the literature on which mode is the most relevant, which might be related to the fact that the most relevant mode is dependent on clinical indication and use. This review only highlights the most probable mechanisms explained in the literature. Differences in opinion regarding the mode of action can also extend to whether a compound acts as a contact‐killer or prevents bacterial adhesion, as seen with guanidine [79, 87]. Potentially, this could be related to the difficulty of separating bacterial adhesion and contact‐killing mechanisms since dead bacteria do not adhere to a matrix. Most studies in this review also do not research the antimicrobial properties of the compounds, instead citing other studies that did, if they mention the mode of action at all. Understanding the mode of action of a compound could give important guidance on how to improve its antimicrobial activity. As an example, most studies agree that QACs penetrate the bacterial cell wall with their aliphatic chain, which is why the length of the chains matters greatly for their antimicrobial results [47].

Obtaining approval to introduce new antimicrobial materials to the market requires extensive research. In this review, we found that 62.2% of all studies did not test for biocompatibility. Furthermore, only 9.6% of the studies conducted any in vivo testing, and a mere 4.4% performed in vivo antimicrobial testing. Considering all the additional limitations and missing data that were already explained, it can be concluded that the absence of the antimicrobial compounds discussed in this review from clinical use may be attributed less to limitations in material design and more to methodological challenges in their evaluation and translation.

The field of antimicrobial polyurethanes is extensive, and several studies have sought to summarize recent advances in this area. To the best of the authors′ knowledge, no comprehensive systematic review focusing on antimicrobial polyurethanes has been published to date; however, several narrative reviews and overviews addressing this or closely related topics are available. Kasi et al. [209] review polyurethane‐based composites exhibiting promising antibacterial properties, providing a valuable overview of polyurethane synthesis, processing methods, and cutting‐edge antibacterial modification strategies. Nevertheless, their work does not constitute a comprehensive systematic review, and the discussion of results and future perspectives remains relatively brief. Wang et al. [210] presented an overview of functionally modified polyurethanes for rendering surfaces antimicrobial, whereas Saleemi et al. [167] discussed antimicrobial polyurethane‐based nanocomposite materials. As narrative overviews, these studies selectively highlight representative literature and provide limited critical or systematic analysis of the available data. Therefore, this systematic review is aimed at providing a comprehensive and detailed assessment of organic antimicrobial modification strategies for polyurethanes. Rather than focusing exclusively on cutting‐edge technologies, the review encompasses all relevant modification approaches reported over the past 20 years to capture broader methodological and developmental trends. In contrast to previous reviews, this work also identifies the limited clinical translation of these technologies as being largely attributable to inconsistencies in testing methodologies and reporting standards.

Previous reviews and overviews have highlighted the need for a more profound understanding of bacteria–PU interactions [209] and have emphasized the importance of further research into dual‐function synergistic antimicrobial systems [167, 210]. They do not adequately address the lack of clinical translation that, as discussed in this review, is likely related to inconsistent reporting practices and regulatory hurdles. In addition, the rapidly increasing bacterial resistance has largely been overlooked, despite projections estimating that antimicrobial resistance could account for up to 10 million deaths annually by 2050 [6, 7]. The escalating resistance underlines the urgent need to develop antibacterial strategies that do not rely on conventional antibiotics. In this context, antimicrobial systems employing synergistic mechanisms are expected to become increasingly important, as they may enable effective eradication of even resistant bacterial strains. Consequently, research efforts in these areas are anticipated to intensify as the global burden of antimicrobial resistance continues to rise.

## 5. Conclusions

For antimicrobial polyurethanes to advance toward clinical application as medical devices, comprehensive in vivo evaluations and extensive biocompatibility assessments are essential. At this moment, the number of antimicrobial polyurethanes in approved medical devices is still low, and with the threat of rising infection incidence and antimicrobial resistance, it is important to develop more alternatives to antibiotics in clinical practice.

This review demonstrates that the field of antimicrobial polyurethanes is vast. Unfortunately, although the result of research is promising, with demonstrated antimicrobial performance of up to (8–9‐log) [36] and (6–7‐log) [33] in 5 min in vitro and 3 log [70] in vivo, there are still big challenges slowing down clinical implementation. These obstacles appear to stem not from a lack of effective technologies but rather from inconsistent reporting practices and regulatory hurdles. To bridge this translational gap, protocols, research, and reporting need to adhere to high‐quality standards and be standardized for both in vitro and in vivo models, thereby enabling more consistent evaluation and regulatory approval of antimicrobial polyurethane systems.

Contact killing antimicrobial polymers, including polyurethanes, must disrupt microbial membranes without adversely affecting human cells. Furthermore, there is a need to demonstrate true nonleaching behavior of the antimicrobial moieties. When such biomaterials are in contact with blood, a further safety requirement is ensuring good hemocompatibility, ensuring thrombosis, coagulation, or hemolysis are not triggered (unless explicitly required). This means that tests closely related to the medical device application and that reflect the duration in the body are important steps in preclinical in vivo studies en route to achieving regulatory approval.

## Funding

This study was supported by the DARTBAC project of the research program NWA‐ORC, which is (partly) financed by the Dutch Research Council (NWO) (NWA.1292.19.354).

## Disclosure

This publication is part of the DARTBAC project (with Project Number NWA.1292.19.354) of the research program NWA‐ORC, which is (partly) financed by the Dutch Research Council (NWO).

## Conflicts of Interest

The authors declare no conflicts of interest.

## Data Availability Statement

Data sharing is not applicable to this article as no datasets were generated or analyzed during the current study.

## Supporting information


**Supporting Information** Additional supporting information can be found online in the Supporting Information section. The protocol for this systematic review was made on February 18, 2025 as outlined in the PRISMA checklist and is provided in the supporting information.
