# Supplementary material for: Organic Polyurethane Surface Structures to Manufacture Noneluting Antimicrobial Polyurethanes: A Systematic Review of Literature
Source: Biomed Res Int. 2026 May 20;2026:6901867. doi: 10.1155/bmri/6901867 (PMC13189482; doi:10.1155/bmri/6901867)
Supplement: Supplementary file 1 — Supporting Information Additional supporting information can be found online in the Supporting Information section. The protocol for this systematic review was made on February 18, 2025 as outlined in the PRISMA checklist and is provided in the supporting information. [file BMRI-2026-6901867-s001.docx]

**“Systematic review protocol - February 18th 2025”**

**Title:** Organic polyurethane surface structures to manufacture non-eluting antimicrobial polyurethanes: A systematic literature review

**Objectives:**

1. Identify the production method, composition and physical and chemical properties of polyurethanes with antimicrobial properties based on anti-adhesion, contact killing abilities.
2. Determine the mode of action and effectiveness of antimicrobial material properties.

**Research question:** "What is the state of the art for anti-adhesion, contact killing, and eluting systems in antimicrobial polyurethanes?"

**Main concepts and synonyms:**

| **Polyurethanes** | **AND** | **Antibacterial** | **AND** | **Anti-adhesion/contact killing/eluting systems** |
| --- | --- | --- | --- | --- |
| Polyurethan*  Polycarbamat* |  | antibacterial  antimicrobial |  | Adhes*  fouling  **OR**  Contact killing  Contact  **OR**  Drug-Eluting  Elut* |

**Exclusion criteria:**

*The search is limited to English texts

*The search is limited to the years 2003-2023

*The search is limited to citations with full texts attached

*The search is limited to published articles

*In further examination all articles on antifungal or anti-viral mechanisms instead of an antibacterial mechanism will be removed

*In further examination all articles on materials that are not based on polyurethanes will be removed

*Articles about applications outside of the medical field will be removed

*In further examination all articles without in vitro or in vivo experiments will be removed

**Table I.** Search reporting

| **Date** | **Person Conducting the Search** | **Database** | **Interface** | **Search Strategy** | **Downloaded citations** |
| --- | --- | --- | --- | --- | --- |
| 18.02.25 | BJH | PubMed | Advanced Search | ("polyurethan*"[Title/Abstract] OR "Polycarbamat*"[Title/Abstract] OR "polyurethanes"[MeSH Terms]) **AND** (“antibacterial"[Title/Abstract] OR "antimicrobial"[Title/Abstract] OR "Anti-Infective Agents"[MeSH Terms]) **AND** ("Elut*"[Title/Abstract] OR "Drug-Eluting"[Title/Abstract] OR Drug-Eluting Stent[MeSH Terms] OR "Contact killing"[Title/Abstract] OR "Contact"[Title/Abstract] OR "Adhes*"[Title/Abstract] OR Bacterial Adhesion[MeSH Terms]OR "fouling"[Title/Abstract]) NOT (“marine"[Title/Abstract] OR “packaging"[Title/Abstract] OR “waste water"[Title/Abstract])  Filters: English Language, Full Text, year: 2003-2025 | 326 |
|  | BJH | Scopus | Advanced Search | ( TITLE-ABS-KEY ( polyurethan* OR polycarbamat* ) **AND** TITLE-ABS-KEY (antibacterial OR antimicrobial ) **AND** TITLE-ABS-KEY (elut* OR drug-Eluting OR ”contact killing”OR contact OR adhes* OR fouling) ) **AND** NOT ( TITLE-ABS-KEY ( marine OR packaging OR "waste water" ) )  Filters: English Language, Article, Final, Full Text, year: 2003-2023 | 621 |
|  | BJH | Web of Science | Advance search | (TS=Polycarbamat* OR TS=polyurethan*) **AND** (TS=antibacterial OR TS=antimicrobial) **AND** (TS=Elut* OR TS=Drug-Eluting OR TS="Contact killing" OR TS=Contact OR TS=Adhes* OR TS=fouling) **NOT** (TS=marine OR TS=packaging OR TS="waste water")  Filters: English Language, Article, year: 2003-2025 | 730 |
|  | BJH | Embase | OVID Advanced search | ((polyurethan* OR Polycarbamat*).ab,ti,kf. or polyurethane/ or polyurethane foam/) **AND** ((antibacterial or antimicrobial ).ab,ti,kf. or antiinfective agent/) **AND** ((Elut* or Drug-eluting or Contact or Adhes* or fouling).ab,ti,kf. or drug eluting stent/ or bacterium adherence/) **NOT** (marine OR packaging OR "waste water").ab,ti,kf.  Filters: English Language, Article, year: 2003-2025 | 283 |
|  | MV | PubMed | Advanced Search | ((("Polyurethane"[Title/Abstract] OR "polyurethan*"[Title/Abstract] OR "polycarbamat*"[Title/Abstract] OR "polyurethanes"[MeSH Terms]) AND ("Antibacterial"[Title/Abstract] OR "Antimicrobial"[Title/Abstract] OR "anti-infective agents"[MeSH Terms]) AND ("Eluting"[Title/Abstract] OR "elut*"[Title/Abstract] OR "drug eluting stents"[MeSH Terms] OR "Drug-eluting"[Title/Abstract] OR ("Contact killing"[Title/Abstract] OR "Contact"[Title/Abstract]) OR ("adhes*"[Title/Abstract] OR "bacterial adhesion"[MeSH Terms]) OR "fouling"[Title/Abstract])) NOT ("marine"[Title/Abstract] OR "packaging"[Title/Abstract] OR "waste water"[Title/Abstract]))  Filters: English Language, Full Text, year: 2003-2023 | 326 |
|  | MV | Scopus | Advanced Search | ( ( ( TITLE-ABS-KEY ( polyurethane ) ) OR ( TITLE-ABS-KEY ( polyurethan* ) ) OR ( TITLE-ABS-KEY ( polycarbamat* ) ) ) AND ( ( TITLE-ABS-KEY ( antibacterial ) ) OR ( TITLE-ABS-KEY ( antimicrobial ) ) ) AND ( ( ( TITLE-ABS-KEY ( eluting ) ) OR ( TITLE-ABS-KEY ( elut* ) ) OR ( TITLE-ABS-KEY ( "Drug-Eluting" ) ) ) OR ( ( TITLE-ABS-KEY ( "Contact killing" ) ) OR ( TITLE-ABS-KEY ( contact ) ) ) OR ( ( TITLE-ABS-KEY ( adhes* ) ) OR ( TITLE-ABS-KEY ( fouling ) ) ) ) ) AND NOT ( TITLE-ABS-KEY ( marine ) OR TITLE-ABS-KEY ( packaging ) OR TITLE-ABS-KEY ( "waste water" ) )  Filters: English Language, Article, Final, Full Text, year: 2003-2023 | 621 |
|  | MV | Web of Science | Advance search | (TS=(polyurethane) OR TS=(polyurethan*) OR TS=(Polycarbamat*)) AND (TS=(antibacterial) R=OR TS=(antimicrobial)) AND (TS=(Eluting) OR TS=(Elut*) OR TS=("Drug-Eluting") OR TS=("Contact killing") OR TS=(Contact) OR TS=(Adhes*) OR TS=(fouling)) NOT (TS=marine OR TS=packaging OR TS="waste water")  Filters: English Language, Article, year: 2003-2023 | 730 |
|  | MV | Embase | OVID Advanced search | ((Polyurethane or polyurethan* OR polycarbamat*).ab,ti,kf. OR polyurethane/ OR polyurethane foam/ AND (antibacterial OR antimicrobial).ab,ti,kf. OR antiinfective agent/ AND (eluting OR Elut* OR “Drug-eluting”).ab,ti,kf. OR drug eluting stent/ OR (“contact killing OR contact).ab,ti,kf. OR (Adhes* OR fouling).ab,ti,kf. OR bacterium adherence/ ) NOT (marine or packaging or "waste water").ab,ti,kf.  Filters: English Language, Article, year: 2003-2023 | 283 |

**Search Strategy PubMed**

**Table II.** Search Strategy PubMed_BJH (Date: 19-02-2025)

| **#** | **Searches** | **Results** |
| --- | --- | --- |
| 1 | polyurethane | 22 363 |
| 2 | polyurethan* | 22 419 |
| 3 | Polycarbamat* | 24 |
| 4 | Polyurethanes[MeSH Terms]^[[1]](#endnote-1)^ | 11 186 |
| 5 | #2 OR #3 OR #4 | 22 442 |
| 6 | antibacterial | 932 020 |
| 7 | antimicrobial | 2 086 626 |
| 8 | Anti-Infective Agents[MeSH Terms] | 885 742 |
| 9 | #6 OR #7 OR #8 | 2 123 890 |
| 10 | Eluting | 93 757 |
| 11 | Elut* | 96 719 |
| 12 | Drug-Eluting | 22 231 |
| 13 | Drug-Eluting Stent[MeSH Terms] | 14 256 |
| 14 | #11 OR #12 OR #13 | 96 719 |
| 15 | "Contact killing" | 303 |
| 16 | Contact | 539 067 |
| 17 | #15 OR #16 | 539 067 |
| 18 | Adhes* | 428 694 |
| 19 | Bacterial Adhesion[MeSH Terms] | 21 674 |
| 20 | fouling | 11 225 |
| 21 | #18 OR #19 OR #20 | 438 539 |
| 22 | #14 OR #17 OR #21 | 1 043 325 |
| 23 | #5 AND #9 AND #22 | 397 |
| 24 | #23 NOT (“marine"[Title/Abstract] OR “packaging"[Title/Abstract] OR “waste water"[Title/Abstract]) | 376 |

**Search Strategy PubMed**

**Table III.** Search Strategy PubMed_MV (Date: 18-02-2025)

| **#** | **Searches** | **Results** |
| --- | --- | --- |
| 1 | Polyurethane | 22 362 |
| 2 | Polyurethan* | 22 418 |
| 3 | Polycarbamat* | 24 |
| 4 | Polyurethanes[MeSH Terms] | 11 186 |
| 5 | #2 OR #3 OR #4 | 22 441 |
| 6 | antibacterial | 931 973 |
| 7 | antimicrobial | 2 086 530 |
| 8 | Anti-Infective Agents[MeSH Terms] | 885 695 |
| 9 | #6 OR #7 OR #8 | 2 123 782 |
| 10 | Eluting | 93 753 |
| 11 | Elut* | 96 715 |
| 12 | Drug-Eluting | 22 230 |
| 13 | Drug-Eluting Stent[MeSH Terms] | 14 255 |
| 14 | #10 OR #11 OR #12 OR #13 | 96 715 |
| 15 | "Contact killing" | 303 |
| 16 | Contact | 539 021 |
| 17 | #16 OR #17 | 539 021 |
| 18 | Adhes* | 428 666 |
| 19 | Bacterial Adhesion[MeSH Terms] | 21 673 |
| 20 | #19 OR #20 | 428 666 |
| 21 | fouling | 11 225 |
| 22 | #15 OR # 18 OR #21 OR #22 | 949 175 |
| 23 | #5 AND #9 AND #22 | 397 |
| 24 | #23 NOT (“marine"[Title/Abstract] OR “packaging"[Title/Abstract] OR “waste water"[Title/Abstract]) | 376  With filters: 326 |

**Table IV.** Search Strategy Scopus_BJH (Date: 20-02-2025)

| **#** | **Searches** | **Results** |
| --- | --- | --- |
| 1 | polyurethane | 94 153 |
| 2 | polyurethan* | 98 958 |
| 3 | Polycarbamat* | 53 |
| 4 | #2 OR #3 | 99 004 |
| 5 | antibacterial | 293 115 |
| 6 | antimicrobial | 454 302 |
| 7 | #5 OR #6 | 642 761 |
| 8 | Eluting | 43 545 |
| 9 | Elut* | 166 033 |
| 10 | Drug-Eluting | 32 365 |
| 11 | #9 OR #10 | 166 033 |
| 12 | "Contact killing" | 511 |
| 13 | Contact | 1 463 570 |
| 14 | #12 OR #13 | 1 463 570 |
| 15 | Adhes* | 835 129 |
| 16 | fouling | 57 578 |
| 17 | #15 OR #16 | 888 727 |
| 18 | #11 OR #14 OR #17 | 2 439 379 |
| 19 | #4 AND #7 AND #18 | 827 |
|  | #19 AND NOT (TITLE-ABS-KEY ( marine )  OR  TITLE-ABS-KEY ( packaging )  OR  TITLE-ABS-KEY ( "waste water" ) ) | 749 |

**Table V.** Search Strategy Scopus_MV (18-02-2025)

| **#** | **Searches** | **Results** |
| --- | --- | --- |
| 1 | polyurethane | 94 129 |
| 2 | polyurethan* | 98 934 |
| 3 | Polycarbamat* | 53 |
| 4 | #1 OR #2 OR #3 | 98 980 |
| 5 | antibacterial | 293 009 |
| 6 | antimicrobial | 454 071 |
| 7 | #5 OR #6 | 642 471 |
| 8 | Eluting | 43 540 |
| 9 | Elut* | 166 018 |
| 10 | ”Drug-Eluting” | 32 362 |
| 11 | #8 OR #9 OR #10 | 166 018 |
| 12 | "Contact killing" | 511 |
| 13 | Contact | 1 463 296 |
| 14 | #12 OR #13 | 1 463 296 |
| 15 | Adhes* | 834 990 |
| 16 | fouling | 57 554 |
| 17 | #15 OR #16 | 888 566 |
| 18 | #11 OR #14 OR #17 | 2 438 936 |
| 19 | #4 AND #7 AND #18 | 826 |
| 20 | #19 AND NOT TITLE-ABS-KEY ( marine )  OR  TITLE-ABS-KEY ( packaging )  OR  TITLE-ABS-KEY ( "waste water" ) | 748  With filters: 621 |

**Table VI.** Search Strategy Web of Science_BJH (Date: 19-02-2025)

| **#** | **Searches** | **Results** |
| --- | --- | --- |
| 1 | polyurethane | 73 031 |
| 2 | polyurethan* | 73 607 |
| 3 | Polycarbamat* | 41 |
| 4 | #2 OR #3 | 73 643 |
| 5 | antibacterial | 238 458 |
| 6 | antimicrobial | 442 298 |
| 7 | #5 OR #6 | 581 102 |
| 8 | Eluting | 70 324 |
| 9 | Elut* | 118 419 |
| 10 | Drug-Eluting | 24 169 |
| 11 | #9 OR #10 | 118 419 |
| 12 | "Contact killing" | 459 |
| 13 | Contact | 1 115 158 |
| 14 | #12 OR #13 | 1 115 158 |
| 15 | Adhes* | 617 649 |
| 16 | fouling | 48 850 |
| 17 | #15 OR #16 | 662 322 |
| 18 | #11 OR #14 OR #17 | 1 824 454 |
| 19 | #4 AND #7 AND #18 | 914 |
|  | #19 NOT (TS=marine OR TS=packaging OR TS="waste water") | 819 |

**Table VII.** Search Strategy Web of Science_MV (Date: 19.02.2025)

| **#** | **Searches** | **Results** |
| --- | --- | --- |
| 1 | polyurethane | 72 578 |
| 2 | polyurethan* | 72 796 |
| 3 | Polycarbamat* | 41 |
| 4 | #1 OR #2 OR #3 | 72 832 |
| 5 | antibacterial | 237 503 |
| 6 | antimicrobial | 382 813 |
| 7 | #5 OR #6 | 523 706 |
| 8 | Eluting | 70 283 |
| 9 | Elut* | 24 062 |
| 10 | “Drug-Eluting” | 459 |
| 11 | #8 OR #9 OR #10 | 118 134 |
| 12 | "Contact killing" | 459 |
| 13 | Contact | 1 090 760 |
| 14 | #12 OR #13 | 1 090 760 |
| 15 | Adhes* | 608 178 |
| 17 | fouling | 47 991 |
| 18 | #15 OR #16 | 652 058 |
| 19 | #11 OR #14 OR #17 | 1 790 924 |
| 20 | #4 AND #7 AND #18 | 914 |
|  | #20 NOT (TS=marine OR TS=packaging OR TS="waste water") | 819  With filters:731 |

**Table VIII.** Search Strategy Embase.ovid _BJH (Date: 19-02-2025)

| **#** | **Searches** | **Results** |
| --- | --- | --- |
| 1 | polyurethane | 18 150 |
| 2 | polyurethan* | 26 077 |
| 3 | polycarbamat* | 21 |
| 4 | polyurethan/ OR polyurethane foam/ | 21 243 |
| 5 | #2 OR #3 OR #4 | 26 098 |
| 6 | antibacterial | 197 952 |
| 7 | antimicrobial | 353 793 |
| 8 | antiinfective agent/ | 227 334 |
| 9 | #6 OR #7 OR #8 | 594 613 |
| 10 | Eluting | 59 143 |
| 11 | Elut* | 145 796 |
| 12 | Drug-Eluting | 46 320 |
| 13 | drug eluting stent/ | 34 699 |
| 14 | #11 OR #12 OR #13 | 145 796 |
| 15 | "Contact killing" | 298 |
| 16 | Contact | 541 903 |
| 17 | #15 OR #16 | 541 903 |
| 18 | Adhes* | 583 974 |
| 19 | bacterium adherence/ | 26 429 |
| 20 | fouling | 14 730 |
| 21 | #18 OR #19 OR #20 | 608 920 |
| 22 | #5 AND #9 AND #21 | 502 |
| 23 | #23 NOT (marine or packaging or “waste water”).ab,ti,kf. | 483 |

**Table IX.** Search Strategy Embase.ovid _MV (19-02-2025)

| **#** | **Searches** | **Results** |
| --- | --- | --- |
| 1 | polyurethane | 18 744 |
| 2 | polyurethan* | 26 077 |
| 3 | Polycarbamat* | 21 |
| 4 | polyurethan/ or polyurethane foam/ | 21 243 |
| 5 | #1 OR #2 OR #3 OR #4 | 26 098 |
| 6 | antibacterial | 197 952 |
| 7 | antimicrobial | 353 793 |
| 8 | antiinfective agent/ | 227 334 |
| 9 | #6 OR #7 OR #8 | 594 613 |
| 10 | Eluting | 59 143 |
| 11 | Elut* | 145 796 |
| 12 | “Drug-Eluting” | 46 320 |
| 13 | drug eluting stent/ | 34 699 |
| 14 | #10 OR #11 OR #12 OR #13 | 145 759 |
| 15 | "Contact killing" | 298 |
| 16 | Contact | 541 903 |
| 17 | #15 OR #16 | 541 903 |
| 18 | Adhes* | 583 974 |
| 19 | bacterium adherence/ | 26 429 |
| 20 | fouling | 14 730 |
| 21 | #18 OR #19 OR #20 | 608 920 |
| 22 | #14 OR #17 OR #21 | 1 260 492 |
| 23 | #5 AND #9 AND #22 | 502 |
| 24 | #23 NOT (marine or packaging or “waste water”).ab,ti,kf. | 483  With filters 283 |

1. Both PubMed and Embase Ovid enable searching with Medical Subject Headings (MeSH) Terms. In PubMed those terms are labeled with [MeSH Terms] in Embase Ovid with a / at the end. Both labels have been used in this protocol. Example: Polyurethanes[MeSH Terms] or polyurethan/ [↑](#endnote-ref-1)
